# An efficient machine-learning framework for predicting protein post-translational modification sites

**DOI:** 10.1038/s41598-025-13178-x

**Published:** 2025-08-25

**Authors:** Heba M. Elreify, Fathi E. Abd El-Samie, Moawad I. Dessouky, Hanaa Torkey, Said E. El-Khamy, Wafaa A. Shalaby

**Affiliations:** 1 Department of Electronics and Electrical Communications Engineering, Faculty of Electronic Engineering, Menoufia University, Menouf 32952, Egypt; 2 Department of Computer Science and Engineering, Faculty of Electronic Engineering, Menoufia University, Menouf 32952, Egypt; 3 Department of Electrical Engineering, Alexandria University, Alexandria, Egypt; 4 Department of Information Technology, College of Computer and Information Sciences, Princess Nourah Bint Abdulrahman University, P.O. Box 84428, Riyadh, 11671 Saudi Arabia

**Keywords:** Post-translational modification, Lysine 2-hydroxyisobutyrylation, Machine learning, LightGBM, Protein language models, ESM, Mutual information, Post-translational modifications, Biomedical engineering

## Abstract

Post-Translational Modifications (PTMs), particularly lysine 2-hydroxyisobutyrylation (Khib), represent critical regulatory mechanisms governing protein structure and function, with mounting evidence underscoring their important implications in cellular metabolism, transcriptional regulation, and pathological processes. Despite this significance, the experimental identification of Khib sites remains constrained by resource-intensive methodologies and the transient nature of these modifications. To overcome these limitations, we introduce HyLightKhib, a computational framework that leverages Light Gradient Boosting Machine architecture for accurate Khib site prediction. Our approach depends on a hybrid feature extraction strategy, integrating Evolutionary Scale Modeling (ESM-2) embeddings with comprehensive Composition, Transition, and Distribution (CTD) descriptors as well as curated amino acid physicochemical properties for fixed-length peptides of 43 amino acids. The proposed classifier demonstrated considerable performance over contemporary algorithms, including XGBoost and CatBoostimplementations through mutual information-based feature selection optimization. Cross-species validation on diverse organisms including, human, parasite , and rice achieved improved Area Under the Receiver Operating Characteristic Curve (AUC-ROC) scores of 0.893, 0.876, and 0.847, respectively, outperforming existing predictors, such as DeepKhib, and ResNetKhib. HyLightKhib represents an advancement in computational PTM prediction, providing enhanced predictive performance and valuable biological insights with direct implications for functional proteomics and PTM-targeted therapies.

**Supplementary Information:**

The online version contains supplementary material available at 10.1038/s41598-025-13178-x.

## Introduction

The PTMs represent a diverse set of chemical changes that occur on proteins after translation, critically regulating protein structure, activity, localization, and interactions ^1^. These modifications greatly expand proteome complexity beyond what the genome encodes, creating a complex regulatory layer that enables dynamic cellular responses to environmental stimuli ^2^. The number of known PTMs continues to expand, with over 200 distinct types identified to date, each with unique biochemical characteristics and functional implications ^3^.

The major categories of PTMs include phosphorylation (addition of phosphate groups to serine, threonine, or tyrosine residues), which regulates the enzymatic activity and signal transduction; glycosylation (attachment of carbohydrate moieties), critical for protein folding and cell-cell recognition; acetylation (addition of acetyl groups), which modulates chromatin structure and gene expression; methylation (addition of methyl groups), involved in epigenetic regulation; ubiquitination (conjugation of ubiquitin proteins), which targets proteins for degradation or alters their subcellular localization; and SUMOylation (attachment of small ubiquitin-like modifiers), which regulates nuclear transport and transcription factor activity ^2,4^. More recently, a growing family of lysine acylations has been identified, including propionylation, butyrylation, crotonylation, succinylation, and 2-hydroxyisobutyrylation, expanding our understanding of how metabolic state influences protein function through PTMs.

Lysine residues are particularly prominent targets for PTMs due to their charged side chains and nucleophilic properties. Among these lysine-specific modifications, 2-hydroxyisobutyrylation (Khib) has emerged as a particularly significant acylation with profound implications for chromatin dynamics, transcriptional regulation, and metabolic control ^5^. First discovered in the histone proteins, Khib adds a bulky 2-hydroxyisobutyryl group (+ 86 Da) that neutralizes lysine positive charge, potentially creating binding sites for regulatory proteins, while disrupting electrostatic interactions ^6^.

Subsequent research has demonstrated that Khib extends beyond histones to numerous non-histone proteins across evolutionarily diverse organisms, from humans to parasites and plants ^7,8^. The conservation of this modification across species underscores its fundamental importance in cellular processes, including metabolism, stress response, and development. Furthermore, recent studies have implicated aberrant Khib patterns in pathological conditions, including cancer, neurodegenerative disorders, and metabolic diseases ^9^, highlighting its potential clinical relevance in precision medicine approaches.

The identification of PTM sites through experimental methods, such as mass spectrometry, remains challenging due to the transient nature of modifications, low modification abundance, and resource-intensive workflows ^10^. These technical limitations have necessitated the development of computational approaches to complement experimental techniques, enabling high-throughput screening of potential modification sites across proteomes.

Computational PTM site prediction faces several key challenges. First, the vast search space of potential modification sites (all candidate residues in the proteome) makes exhaustive experimental screening prohibitively expensive and time-consuming. Second, the subtle sequence patterns distinguishing modified residues from unmodified ones often involve complex, non-linear relationships that extend beyond simple sequence motifs ^11^. Third, the biological context in which PTMs occur varies significantly across tissues, developmental stages, and environmental conditions, necessitating algorithms with strong generalization capabilities. Finally, the evolutionary divergence in PTM patterns across species requires computational approaches that can adapt to organism-specific characteristics, while leveraging conserved features ^12^.

The computational prediction of PTM sites has evolved significantly over the past two decades, progressing through several methodological paradigms. Early approaches relied primarily on sequence-based features and simple statistical models. For instance, the Group-based Prediction System (GBS) used position-specific scoring matrices to identify kinase-specific phosphorylation sites ^13^. On the other hand, tools like FSL-Kla developed a few-shot learning hybrid framework integrating multiple features for lactylation site prediction ^14^.

As the field advanced, machine learning approaches incorporating more complicated features gained prominence. LFPred implements a K-nearest neighbor classification algorithm integrated with multiple sequence-derived features to identify lysine malonylation sites ^15^. Similarly, tools like iPTM-mLys ^16^ and PTMPred leveraged support vector machines and random forests with combinations of sequence-based, structural, and evolutionary features to predict various lysine modifications.

In recent years, gradient boosting techniques, particularly Light Gradient Boosting Machine (LightGBM), have gained traction in PTM site prediction due to their ability to model intricate feature interactions, while maintaining computational efficiency. Ahmad et al. introduced Mal-light ^17^, which combined evolutionary features with LightGBM to improve the prediction of lysine malonylation sites, achieving notable gains in both accuracy and processing speed. Similarly, LightGBM was applied with a multi-feature fusion strategy for propionylation site prediction, reporting enhanced predictive performance ^18^. The efficacy of LightGBM was further substantiated by Arafat et al., who used sequential bi-peptide evolutionary features to predict glutarylation sites with high accuracy ^19^. Additionally, Shovan et al. demonstrated that LightGBM, coupled with evolutionary features, effectively addressed class imbalance, while improving glutarylation site prediction ^20^. Collectively, these studies underscore the growing prominence of LightGBM in PTM prediction, highlighting its strong predictive capabilities and computational advantages.

The most recent paradigm shift has been directed toward deep learning methodologies that can automatically extract relevant features from raw sequence data. BERT-Kcr represents this approach by utilizing a pre-trained Bidirectional Encoder Representation from Transformers (BERT) architecture for lysine crotonylation site prediction ^21^. Similarly, for lysine acetylation prediction, a cascade classifier approach based on Complex-Valued Polynomial Models (CVPMs) has been developed, incorporating sequence and structural features to effectively address the challenge of imbalanced datasets ^22^. Similar deep-learning frameworks have been developed for other PTMs, including DeepPhos for phosphorylation ^23^ and DeepUbi for ubiquitination ^24^.

For Khib specifically, several predictors have been proposed. Early models like iLys-Khib ^25^ and KhibPred ^26^ have utilized sequence-based features, position-specific amino acid propensity, and physicochemical properties to predict Khib sites, achieving a reasonable accuracy. The 2-hydr_Ensemble approach has been proposed, employing bi-profile Bayesian statistical features with an ensemble classification algorithm to identify Khib sites across multiple organisms ^27^. More recently, deep learning advancements have introduced methods such as DeepKhib ^6^, which integrates a Convolutional Neural Network (CNN) with one-hot encoding; ResNetKhib ^28^, which combines one-dimensional CNN with transfer learning; and DeepKpred ^29^, which employs an ensemble model consisting of both CNN and LSTM architectures.

While these existing predictors demonstrate superior performance, significant opportunities for improvement exist. As Khib represents a relatively recently discovered PTM, the field faces inherent challenges, including an evolving understanding of the modification biological mechanisms and insufficient exploration of optimal computational frameworks specifically tailored for this PTM. Although Khib sites have been increasingly identified across various organisms, developing computational frameworks that can effectively learn from these data and generalize across species remains a significant challenge. Furthermore, the computational intensity of some current approaches, which typically demand extensive resources and thousands of training epochs, limits their practical applicability in high-throughput research environments.

To address the limitations of existing approaches, this study presents HyLightKhib, a computational framework that integrates protein language model embeddings (ESM-2), sequence-based descriptors (CTD), and physicochemical properties (AAindex) to capture complementary features relevant to lysine 2-hydroxyisobutyrylation (Khib) site prediction. The term “Hy” in HyLightKhib reflects the hybrid nature of these integrated features. The key contributions of this work are as follows:

- A unified representation strategy that captures evolutionary, sequence, and physicochemical characteristics of Khib sites by combining ESM-2 embeddings, CTD descriptors, and AAindex features.
- Application of mutual information-based selection to retain the most informative features, thereby reducing redundancy and enhancing computational efficiency.
- Attainment of significantly reduced training times (16–528 times faster) , lower memory usage (10–127 times less), and higher inference speed (24–4677 times faster) relative to existing deep learning methods, while maintaining competitive predictive performance.
- Demonstration of consistent performance across taxonomically diverse species (human, parasite, and plant), highlighting the framework potential applicability in biomedical research, agricultural biotechnology, and drug discovery.

The remainder of this paper is organized as follows. Sect."Methods" presents the methodological framework; Sect."Results and discussion" presents comprehensive results and discussion; and Sect."Conclusion" provides the conclusion with future research directions.

**Fig. 1:**
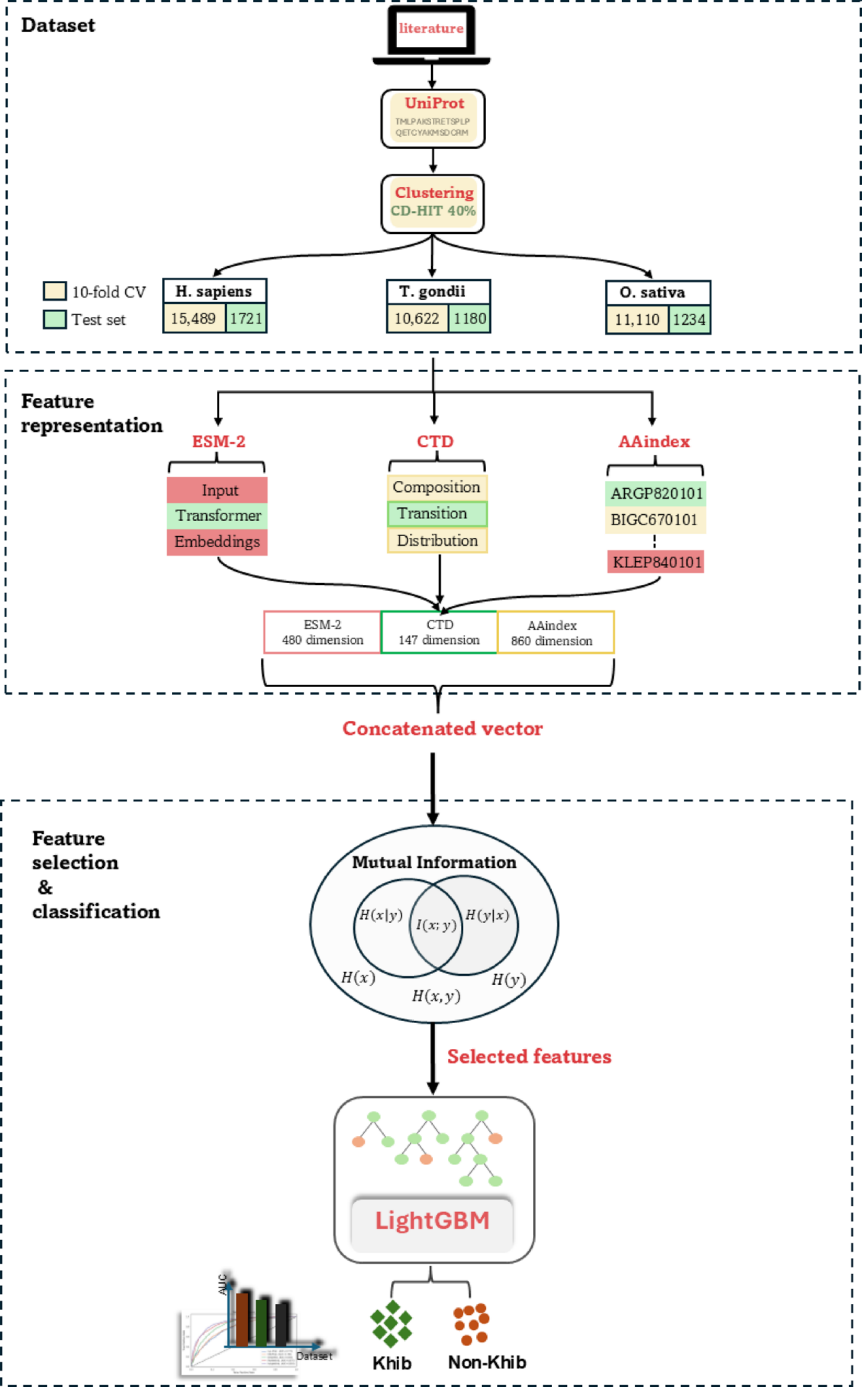
Flowchart of the proposed framework. Overview of the datasets utilized in the study: *H. sapiens*, *T. gondii*, and *O. sativa*, feature representation methods employed include ESM-2, CTD, and AAindex. Feature selection using the mutual information algorithm, and the classification step utilizing the LightGBM classifier to predict binary labels for Khib residues.

## Methods

The proposed framework leverages advanced computational techniques to predict Khib sites. The framework incorporates datasets from three species: *H. sapiens (human)*,* T.gondii (parasite)*, and *O. sativa (Rice)*, with 17,210, 12,344, and 11,802 peptide sequences, respectively. Features were extracted using ESM-2 embeddings, CTD descriptors, and AAindex-derived physicochemical properties. Mutual Information (MI) was employed to enhance performance in feature selection. The LightGBM classifier was used to distinguish Khib-modified residues from non-modified ones. A schematic representation of the flowchart is shown in Fig. 1.

### Dataset

Experimentally verified Khib sites were curated from peer-reviewed publications, where the original authors obtained appropriate ethical approvals, institutional review board permissions, and informed consent as required for their experimental studies for three evolutionarily diverse organisms: *H. sapiens *^10,30,31^, *T. gondii *^32^ and *O. sativa *^33,34^ species. For each organism, Khib-modified sites (positive samples) and their corresponding protein accession numbers were collected from these publications. The initial datasets contain 25,676, 17,594, and 14,079 experimentally-validated Khib sites for *H. sapiens*, *T. gondii*, and *O. sativa*, respectively, distributed across multiple tissue/cell types as detailed in Table 1.

**Table 1:** Distribution of samples in the datasets used for Khib site prediction.

Dataset		Number of positive samples	Number of proteins	Number of positive clusters	Total samples	Ref.
*H. sapiens*	Hela cells	6544	1725	8605	17,210	^10^
	Lung cells	8765	2484			^30^
	Pancreatic cancer cells	10,367	2325			^31^
*T. gondii*	ME49	8092	1720	5901	11,802	^32^
	RH	9502	1950			
*O. sativa*	*O. sativa* seeds	9916	2512	6172	12,344	^33^
	*O. sativa* leaves	4163	1596			^34^

**Fig. 2:**
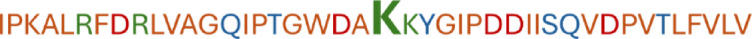
Amino acid sequence of a 43-residue peptide centred on a Khib site.

Rather than retrieving complete protein sequences, we developed a Python ^35^ script that directly extracts peptide sequences of the required length, centred on the Khib-modified lysine residues as represented in Fig. 2. This script utilized the protein accession numbers and specific lysine position information from the published studies to query the UniProt database and extract the target lysine with its surrounding amino acids. This approach aligns with the typical workflow of mass spectrometry-based PTM detection, where proteins are digested into small peptides before analysis.

The optimal window size for peptide extraction was determined through a systematic evaluation procedure. Although window sizes for PTM prediction typically range from 35 to 39 amino acids ^36^, we conducted our optimization experiment due to the limited number of published predictors for Khib sites. We extracted peptides with window sizes ranging from 35 to 47 amino acids (i.e., 17–23 residues flanking the central lysine on each side) and evaluated the model performance for each window length using the *H. sapiens* dataset as a representative model due to its relatively large number of samples, with the AUC averaged over 10-fold cross-validation.

For lysine residues located near protein termini, where insufficient flanking residues were available, the symbol “X” was used as padding to maintain uniform sequence length across all peptides. As shown in Supplementary Table **S1**, the 43-residue window (21 residues on each side of the central lysine) achieved the highest AUC and was chosen as the optimal window for further analyses.

Selecting negative samples (non-Khib-modified lysine residues) is critical for PTM prediction model development. Instead of retrieving complete protein sequences, we developed a Python script to directly extract peptide sequences. We developed a Python script that used accession numbers and known Khib site positions to extract all lysine-centred peptides from the proteins, excluding those at reported modification sites.

This approach yielded a substantially larger pool of potential negative samples compared to positive ones. For instance, in the HeLa cells from the *H. sapiens* dataset, we collected 6,544 positive samples versus 70,828 potential negative samples.

To address the inherent class imbalance while preserving biological relevance, positive and negative peptides were clustered by sequence similarity, after which negative clusters were randomly selected to match the number of positive clusters. This clustering-based balancing strategy yielded a final dataset with a 1:1 positive-to-negative ratio, thereby mitigating class imbalance bias during model training. This balancing methodology aligns with the established PTM prediction studies in general ^37–39^ and the Khib prediction studies specifically ^6^.

The clustering step was implemented using the widely established Cluster Database at High Identity with Tolerance (CD-HIT) tool to reduce sequence redundancy and prevent overfitting. Sequences sharing greater than 40% sequence similarity were grouped into clusters, with one representative sequence retained from each cluster to eliminate redundant training examples. The selection of the 40% similarity threshold is based on established practices in PTM prediction studies ^28,40,41^, where this threshold has been demonstrated to effectively balance dataset diversity while maintaining sufficient training examples for robust model performance.

This clustering approach was applied to the positive and negative peptide sets using CD-HIT with default settings, except for the similarity threshold, which was set to 0.4, and the word length was adjusted accordingly to 2. After clustering, the original 25,676 positive samples from *H. sapiens* were reduced to 8,605 representating positive clusters. Similarly, the *T. gondii* and *O. sativa* datasets were reduced from 17,594 to 5,901 and from 14,079 to 6,172 positive clusters, respectively, demonstrating substantial redundancy removal across all species datasets.

Negative samples underwent the same clustering process. For *H. sapiens*, 257,692 lysine-centred negative peptides were reduced to 31,052 clusters. For *T. gondii*, 62,201 peptides yielded 31,805 clusters, and for *O. sativa*, 89,409 peptides were reduced to 13,347 clusters. From these clusters, we randomly selected an equal number of negative clusters to match the positive samples: 8,605 for *H. sapiens*, 5,901 for *T. gondii*, and 6,172 for *O. sativa*, resulting in final balanced datasets of 17,210, 11,802, and 12,344 peptides, respectively.

For robust model development and evaluation with statistical rigour, each species-specific balanced dataset was partitioned into training (90%) and independent test (10%) sets. This data partitioning strategy ensures reliable performance estimation and prevents overfitting bias that could compromise model generalization. The training set underwent 10-fold cross-validation for model optimization and hyperparameter tuning, a statistical resampling technique that provides stable performance estimates by reducing variance in evaluation metrics and ensuring that model performance is not dependent on a particular training-testing split ratio.

The independent test set was reserved exclusively for the final performance assessment, serving as an unbiased holdout dataset to evaluate true model generalization capacity. This evaluation framework, widely accepted in computational biology and machine learning, provides reliable performance estimates by reducing selection bias and overfitting, thereby increasing confidence in the reported model accuracy.

### Feature representation

Feature representation (encoding) is a critical step in protein sequence analysis, as it translates biological sequences into numerical formats compatible with machine learning algorithms ^42^. The selection of encoding strategies significantly impacts model performance, as these strategies capture the biological properties and patterns underlying sequence data ^43^. To optimize predictive accuracy, we systematically evaluated individual feature representation methods, including ProtBERT ^44^, Word2Vec ^45^, one-hot encoding, and the BLOSUM62 matrix ^46^, along with ESM-2, CTD, and AAindex features. We further explored combinations of these features by integrating all representation methods to enhance prediction performance. Subsequently, we applied an iterative elimination strategy, discarding one feature representation method at a time from the combined set to assess its impact on performance. This process revealed the subset of features that contributed most significantly to model accuracy, as discarding residual features does not degrade performance. The final selected feature set integrates evolutionary insights from ESM-2, sequence-based patterns from CTD, and physicochemical properties from AAindex descriptors. A detailed comparison of alternative feature representation methods is provided in Supplementary Table S2 and Fig. S1.

#### Embedding-based feature representation

The ESM represents a state-of-the-art transformer-based protein language model architecture designed to capture the evolutionary, structural, and functional characteristics inherent in protein sequences ^47^. The ESM-2 model employs a self-supervised learning paradigm, wherein the transformer architecture learns contextualized representations through masked language modeling on large-scale protein sequence databases. Specifically, the model depends on multi-head self-attention mechanisms that enable the simultaneous modeling of local residue interactions and long-range dependencies within protein sequences, thereby capturing both short-range physicochemical properties and distant evolutionary constraints ^48^.

The embedding generation process in ESM-2 follows a hierarchical approach, where each amino acid position is first converted into learnable token embeddings, which are subsequently processed through multiple transformer layers. Each transformer block consists of multi-head self-attention layers followed by position-wise feed-forward networks, with residual connections and layer normalization applied throughout. The self-attention mechanism computes attention weights between all pairs of residues, allowing the model to integrate contextual information from the entire sequence when generating representations for each position. The final hidden states from the last transformer layer serve as contextualized embeddings that encode both local amino acid properties and global sequence context ^49^.

Model selection for this study involved the systematic evaluation of three ESM-2 variants with different parameter scales and embedding dimensions. The esm2_t6_8M_UR50D model (8 million (8 M) parameters, 6 layers, 320-dimensional embeddings) achieved a cross-validation AUC of 0.757, while the larger esm2_t30_150M_UR50D model (150 M parameters, 30 layers, 640-dimensional embeddings) yielded an AUC of 0.759. The intermediate esm2_t12_35M_UR50D model (35 M parameters, 12 layers, 480-dimensional embeddings) demonstrated improved performance with an AUC of 0.762, representing an improvement over smaller and larger variants.

The observed performance pattern reflects the complex relationship between model capacity and task-specific optimization. As the number of parameters in ESM-2 models increases, the embedding dimensionality expands (320 → 480 → 640 dimensions), theoretically providing more informative protein representations through enhanced feature space complexity. However, the marginal performance gains from the largest model relative to the intermediate variant do not justify the substantial increase in computational overhead and memory requirements. The optimal performance of the esm2_t12_35M_UR50D variant shows that 480-dimensional embeddings capture sufficient evolutionary and structural information for Khib prediction tasks, while maintaining computational tractability.

Each peptide sequence was processed through the pre-trained ESM2-35 M model to generate residue-level embeddings, where each amino acid is represented as a 480-dimensional vector that encapsulates multi-scale biological information and captures essential protein sequence properties. However, mean pooling aggregation was applied across all residue-level embeddings within each peptide sequence to reduce the computational complexity. The resulting 480-dimensional feature vectors served as input representations for the Khib prediction pipeline, optimally balancing biological informativeness with computational efficiency.

#### Sequence-based feature representation

Composition, Transition, and Distribution (CTD) features, originally introduced by Dubchak et al. ^50^, are widely utilized in computational biology for encoding protein sequences ^51–53^. These features provide a structured representation of sequence-level characteristics by capturing the physicochemical properties of amino acids. CTD features quantify amino acids’ overall composition, frequency of transitions, and spatial distribution based on their physicochemical attributes, such as hydrophobicity, charge, polarity, and polarizability. For each property, amino acids are categorized into three predefined classes, and each amino acid is encoded with an index of 1,2 or 3 based on its group. For example, in terms of hydrophobicity, amino acids are grouped as follows: group 1 (Polar) includes {R, K, E, D, Q, N}; group 2 (Neutral) includes {G, A, S, T, P, H, Y}; and group 3 (Hydrophobic) includes {C, L, V, I, M, F, W}. This classification framework is critical for calculating CTD descriptors, which are divided into components: Composition, Transition, and Distribution.

** Composition (C-CTD): **The composition descriptor represents the normalized frequency of amino acids belonging to a specific property group within a protein sequence. For a sequence of length 

$\:L$, the composition descriptor of a property group $\:i$ is calculated as.

1 $$\:\begin{array}{c}{C}_{i}=\:\raisebox{1ex}{${n}_{i}$}\!\left/\:\!\raisebox{-1ex}{$L$}\right.\end{array}$$

where $\:{n}_{i}$ is the number of amino acids in the group $\:i$, and $\:L$ is the total sequence length. This descriptor generates an output vector of dimension 21, reflecting the combination of seven amino acid property groups and three physicochemical properties (e.g., hydrophobicity, polarity, and polarizability).

For instance, consider the sequence {AFDQFGHIKLMEPRQTSIWS}v of length $\:L=20$. Using hydrophobicity-based grouping, the counts for each group are as follows: group 1 occupies 6 positions, group 2 occupies 7 positions, and group 3 occupies 7 positions within the sequence. The corresponding composition values are calculated as:

2 $$\:\begin{array}{c}{C}_{group1}={6}\!\left/\:\!{20}\right.=0.3,\:{C}_{group2}={7}\!\left/\:\!{20}\right.=0.35,\:{C}_{group3}={7}\!\left/\:\!{20}\right.=0.35\end{array}$$

This process is repeated for all seven physicochemical properties, resulting in a feature vector of size 21 (7 properties × 3 groups).

**Transition (T-CTD): **The descriptor captures the frequency of transitions between amino acids from different groups within the sequence. A transition occurs when an amino acid from a different group follows an amino acid from a certain group. For a pair of property groups $i$ and $j$, the transition is computed as:

3 $$\:\begin{array}{c}{T}_{\varvec{i}\varvec{j}}=\frac{\left(\text{Number of transitions between groups i and j}\right)}{L-1}\end{array}$$

where $\:L-1$ represents the total number of possible transitions in a sequence of length $\:L$. For instance, in the sequence {AFDQFGHIKLMEPRQTSIWS}, transitions are counted based on group assignments. There are 2 transitions between group 1 and group 2 with $\:L-1=19$, and hence the transition frequency is:

4 $$\:\begin{array}{c}{T}_{\text{group1}\to\:\text{group2}}=\frac{2}{19}=0.105\end{array}$$

This calculation is performed for all pairwise combinations of the three groups across all physicochemical properties, generating a feature vector of size 21.

**Distribution (D-CTD): **The descriptor describes the relative positions of amino acids in each group across the sequence, focusing on the spatial spread of these residues. It identifies the positions of the first, 25th percentile, median (50th percentile), 75th percentile, and last occurrence of amino acids in each group. For a group $i$, the relative position of the $\:{p}^{th}$ occurrence is calculated as.

5 $$\:\begin{array}{c}{D}_{\it{i},\it{p}}=\frac{\text{Position of the}\:{\it{p}}^{\text{th}}\:\text{occurrence of group \it{i}}}{L}\end{array}$$

where $\:p$ represents one of the specified percentiles and $\:L$ is the sequence length. For example, in the sequence {AFDQFGHIKLMEPRQTSIWS} and hydrophobicity-based grouping, 7 residues belong to group 2 (Neutral), and their positions in the sequence are: 1, 6, 7, 13, 16, 17 and 20. The first residue is at position (1), the 25% residue $\:((25/100)\:\times\:7=1.75\approx 2)$ representing the 2nd occurrence is at position 6,  the 50% residue $((50/100) \times 7 = 3.5 \approx 4)$ representing the 4th occurrence is at position 13, the 75% residue $\:((75/100)\:\times\:7=5.25 \approx5)$ representing the 5th occurrence is at position 16, and the last residue $\:((100/100)\:\times\:7=7)$ representing the 7th occurrence is at position 20. Thus, the descriptors for group 2 are:

6 $$\:\begin{array}{c}{D}_{\text{2,1}st}=\frac{1}{20}\:\times\:100=5,{D}_{\text{2,25}\%}=\frac{6}{20}\:\times\:100=30,\:{\:D}_{\text{2,50}\%}=\frac{13}{20}\:\times\:100=65\end{array}$$

7 $$\:\begin{array}{c}{D}_{\text{2,75}\%}=\frac{16}{20}\:\times\:100=80,\:{\:D}_{\text{2,100}\%}=\frac{20}{20}\:\times\:100=100\end{array}$$

These calculations are repeated for all three groups and seven physicochemical properties, producing a total of (7 × 3 × 5) = 105 distribution features.

#### **Property-based feature representation**

The AAindex database ^54^, a comprehensive repository comprising over 566 numerical indices, characterizes diverse physicochemical, biochemical, and structural properties of amino acids, constituting an invaluable resource for protein sequence analysis. In this study, 20 indices were selected for their established relevance to protein functionality, structural characteristics, and potential involvement in PTM mechanisms. These indices are enumerated in Supplementary Table S3. The selection process was guided by a systematic motif analysis of each species-specific dataset using the Two Sample Logo (Sect."Comparative motif analysis and position-specific amino acid preferences"), allowing for dataset-driven feature selection.

The distribution of key amino acid indices, hydrophobicity, residue volume, polarity, net charge, Electron-Ion Interaction Potential (EIIP), and information entropy across the 20 standard amino acids highlights their different contributions to amino acid properties. Hydrophobicity reflects the interaction of residues with the aqueous environment, playing a crucial role in protein folding and binding. Residue volume provides insights into steric effects within the protein structure, while polarity and net charge capture residue-specific interactions, such as hydrogen bonding and electrostatic effects, which are critical for determining the propensity of Khib sites. EIIP quantifies the electronic properties of amino acids, offering a window into their functional roles. Lastly, the entropy of information measures the variability and conservation of amino acids, shedding light on evolutionary constraints that shape sequence functionality. These indices collectively provide a comprehensive physicochemical and structural characterization essential for understanding the determinants of Khib modifications.

The encoding process maps each amino acid in a sequence to its corresponding numerical values for the selected 20 indices, generating a comprehensive numerical vector that preserves both residue-specific and positional information. This results in a feature vector of dimensions $\:L\times20$, where $\:L$ denotes the sequence length. For sequences with $\:L=43,\:$ the resulting feature vector has a dimensionality of 860. This approach ensured the retention of sequence-level and residue-specific characteristics, providing a rich representation of the physicochemical landscape for subsequent machine-learning tasks.

#### Complementary roles of hybrid features in modeling Khib modification mechanisms

This section highlights how integrating different feature types, namely evolutionary, compositional, and physicochemical, enhances the prediction of Khib sites by capturing complementary aspects of protein sequence information:

- ESM-2 evolutionary embeddings; trained on millions of diverse protein sequences from UniRef50 ^55^, provide context-aware representations by leveraging transformer-based attention trained on large protein databases. They capture deep evolutionary conservation, non-local residue interactions, and implicit structural and domain information, enabling detection of complex enzymatic recognition patterns without relying on protein 3D structures ^43,56^.
- CTD descriptors capture local biochemical environments essential for enzymatic activity. Composition features reflect the abundance of amino acids with specific properties, supporting electrostatic interactions favourable for Khib catalysis. Transition and distribution descriptors highlight sequence boundary regions and spatial property distributions important for enzyme-substrate specificity.
- AAindex features encode experimentally-validated physicochemical properties (e.g., hydrophobicity, steric hindrance) of individual amino acids. Unlike CTD, which generalizes properties into broad categories, AAindex provides fine-grained, position-specific numeric values, offering precise biochemical insights into enzymatic recognition conditions.

Together, these hybrid features offer a multifaceted view of Khib modification mechanisms, with each contributing unique and complementary information on evolutionary context from ESM-2, biochemical composition from CTD, and physicochemical precision from AAindex, resulting in more accurate and biologically informed Khib site prediction.

### Mutual information for feature selection

Mutual Information (MI) ^57^ was employed as the feature selection algorithm to enhance model performance and mitigate the risk of overfitting by identifying and prioritizing the most informative features ^58^. MI quantifies the statistical dependency between two random variables by measuring the reduction in entropy of one variable given knowledge of the other ^59^. This capability makes MI highly suitable for complex biological datasets, as it captures both linear and non-linear relationships between features and the target variable. Mathematically, MI for discrete random variables is expressed as:

8 $$\:\begin{array}{c}I\left(x;y\right)=\:\sum\limits_{x \in X}\sum\limits_{y \in Y}p\left(x,y\right)\text{log}\frac{p\left(x,y\right)}{p\left(x\right)p\left(y\right)}\end{array}$$

where $\:X$ represents the feature variable, $Y$ is the target variable, $\:p(x,y)$ denotes their joint probability distribution and $\:p\left(x\right)$ and $\:p\left(y\right)$ are their respective marginal probability distribution. A higher value of $\:I(x;y)$ indicates a stronger dependency between the feature and the target, highlighting its relevance for the classification task.

The choice of MI is based on experimental comparisons with alternative feature selection methods, each based on distinct mathematical principles. ANOVA, a filter-based method ^60^, ranks features based on their statistical relationship with the target variable. Although computationally efficient, ANOVA assumes linear relationships and does not account for interactions or non-linear dependencies among features. Recursive Feature Elimination (RFE), a wrapper-based method ^61^, iteratively trains a model to eliminate the least important features, which allows it to account for feature interactions. However, RFE is computationally expensive, particularly for high-dimensional datasets like those used in this study. *L*_1_regularization (Lasso) ^62^ and Elastic Net embedded methods ^63^ incorporate feature selection during model training by penalizing coefficients of less important features.

In comparison, MI, as a filter-based method, was found to offer distinct advantages in this study. Its ability to capture both linear and non-linear dependencies without requiring iterative model training made it computationally efficient, while still being robust in identifying biologically relevant features. Using the scikit-learn library, MI scores were computed for all features in the dataset to quantify their relevance to the classification of modified versus non-modified lysine residues. Features were ranked in descending order of their MI scores, ensuring that the most statistically informative features are prioritized. To reduce dimensionality while maintaining predictive performance, the top 700 features were selected based on their MI scores. These features provided a rich and concise representation of the dataset, serving as input to the LightGBM classifier for Khib site prediction.

### Light gradient boosting machine (LightGBM)

LightGBM is a state-of-the-art gradient boosting framework developed by Microsoft  ^64^, designed to address key limitations of traditional gradient boosting methods. It introduces several innovative techniques, such as histogram-based learning and Exclusive Feature Bundling (EFB), to enhance computational efficiency and scalability. Unlike conventional level-wise tree growth strategies, LightGBM employs a leaf-wise (best-first) tree growth strategy. This approach prioritizes splitting the leaf with the highest loss reduction, leading to deeper and more accurate trees while reducing training time and memory consumption.

The objective function of LightGBM is a combination of a loss function that measures how well the model fits the data and a regularization term to prevent overfitting by penalizing model complexity. At iteration $\:t$, it can be expressed as:

9 $$\mathcal{O}^{(t)} = \sum_{i=1}^{N} l \left( y_i, \hat{y}_i^{(t-1)} + f_t(x_i) \right) + \Omega(f_t)$$

where $\:N$ is the number of training samples, $\:{y}_{i}$ is the true label for the $\:ith$ sample, $\:\hat{y}_{i}^{\left(t-1\right)}$ is the predicted value at iteration $\:(t-1)$, $\:{f}_{t}\left({x}_{i}\right)$ is the output of the newly added tree at iteration $\:t$, $\:l({y}_{i},\:\hat{y}_{i})$ is the binary cross-entropy (log loss) function and $\:\varOmega\:({f}_{t})$ is the regularization term to control tree complexity, defined as:

10 $$\:\begin{array}{c}\varOmega\:\left({f}_{t}\right)=\:\gamma T+\frac{1}{2}\:\lambda\sum\limits_{j=1}^{T}{{w}_{j}}^{2}\end{array}$$

where $\:T$ is the number of leaf nodes, $\:{w}_{j}$ is the $\:jth$ leaf, γ is a parameter controlling the penalty for adding new leaf nodes, and λ is the $\:{L}_{2}$ regularization term on leaf weights.

At each boosting iteration, LightGBM approximates the loss using a second-order Taylor expansion to enable efficient optimization:

11 $$\mathcal{O}^{(t)} \approx \sum_{i=1}^{N} \left[ g_i f_t(x_i) + \tfrac{1}{2} h_i f_t(x_i)^2 \right] + \Omega(f_t)$$

where $\:{g}_{i}$ and $\:{h}_{i}$ are the first- and second-order gradients, and they are given by:

12 $$g_i = \frac{\partial \, l(y_i, \hat{y}_i)}{\partial \hat{y}_i}$$

13 $$h_i = \frac{\partial^2 \, l(y_i, \hat{y}_i)}{\partial \hat{y}_i^2}$$

Compared to XGBoost ^65^, LightGBM key strengths lie in its memory efficiency and training speed. While XGBoost employs a pre-sorted algorithm and stores all possible split points for continuous features, LightGBM implements histogram-based binning that discretizes continuous features into discrete bins. This optimization significantly reduces memory usage and computational complexity. Additionally, LightGBM Exclusive Feature Bundling (EFB) algorithm effectively manages sparse features by bundling mutually exclusive features, further reducing memory requirements without compromising model accuracy.

When compared to CatBoost ^66^, LightGBM handles categorical features through a different approach. While CatBoost employs ordered target statistics with a permutation-based technique to prevent target leakage and reduce prediction shift, LightGBM depends on a special algorithm for categorical feature splitting based on Gradient-Based One-Sided Sampling (GOSS). GOSS retains instances with large gradients and random samples instances with small gradients, maintaining accuracy while improving training efficiency. However, the CatBoost ordered boosting approach may provide a more robust handling of categorical features in scenarios with high-cardinality categorical variables.

### Evaluation metrics

To ensure a robust and reliable evaluation of the proposed framework, stratified 10-fold cross-validation  ^67^ was employed. This method divides the dataset into 10 equally-sized folds, while preserving the original class distribution within each fold. During each iteration, nine folds were used for training, and the remaining fold was reserved for validation. This approach ensures that all samples are used for both training and validation across iterations. Furthermore, an independent test set comprising 10% of unseen data from each dataset was held out for final performance evaluation, providing an unbiased assessment of the model generalization capabilities to new data.

The model performance was evaluated using multiple complementary metrics derived from the confusion matrix, comprising True Positives (*TP*), True Negatives (*TN*), False Positives (*FP*), and False Negatives (*FN*). The following metrics were computed:

14 $$\:\begin{array}{c}Accuracy\:\left(ACC\right)=\frac{TP+TN}{\left(TP+TN+FP+FN\right)}\end{array}$$

15 $$\:\begin{array}{c}Sensitivity\:\left(SN\right)=\frac{TP}{\left(TP+FN\right)}\end{array}$$

16 $$\:\begin{array}{c}Specificity\:\left(SP\right)=\:\frac{TN}{\left(TN+FP\right)}\end{array}$$

17 $$\:\begin{array}{c}F1=\frac{\left(2\:\times\:PR\:\times SN\right)}{\left(\:PR+SN\right)}\end{array}$$

18 $$\:\begin{array}{c}Precision\:\left(PR\right)=\frac{TP}{\left(TP+FP\right)}\end{array}$$

19 $$\:\begin{array}{c}\:MCC=\frac{\left(TP\times TN\right)-\left(FP\times FN\right)}{\sqrt{\left(TP+FP\right)\left(TP+FN\right)\left(TN+FP\right)\left(TN+FN\right)}}\end{array}$$

Accuracy provides an overall measure of the model predictive correctness, while Sensitivity (also known as Recall in balanced binary classification tasks) is particularly important for capturing the proportion of true positive predictions, ensuring the model ability to identify key positive instances, which is crucial in biological applications like PTM prediction. Specificity gives the model ability to correctly identify negative samples, reducing false positives that could lead to erroneous biological inferences. The F1-Score balances Precision and Sensitivity, offering a harmonized metric suited for scenarios where both false negatives and false positives are of concern. Finally, the Matthews Correlation Coefficient (MCC) is a strong measure of how well the model performs overall. It is especially useful for checking the model reliability in binary classification tasks with balanced datasets, as in our experiments.

To further assess classification performance, Receiver Operating Characteristic (ROC) curves were generated by plotting the True Positive Rate (TPR) against the False Positive Rate (FPR) across varying decision thresholds ^68^. Additionally, the AUC was calculated, offering a threshold-independent evaluation of the model discriminative power. An AUC value close to 1 indicates excellent discriminative ability, while values closer to 0.5 reveal random prediction. Comparative ROC analysis was conducted across all experimental configurations, enabling a quantitative evaluation of model performance and facilitating insight into the effectiveness of different feature encoding methods and classifiers.

Collectively, these evaluation protocols are reinforced by a comprehensive overfitting prevention strategy that ensures robust model generalization. This strategy includes (1) Dataset balancing and redundancy removal: balanced datasets with 1:1 positive-to-negative ratios prevent class imbalance bias, while CD-HIT clustering with 40% sequence similarity threshold eliminates redundant training examples that could lead to memorization rather than pattern learning; (2) Dimensionality optimization: mutual information-based feature selection reduces the risk of overfitting by retaining only the most informative features, while eliminating noise and irrelevant dimensions; (3) Rigorous data partitioning: the combination of stratified 10-fold cross-validation for model development and completely independent test sets (10% holdout) ensures unbiased performance assessment, with test data remaining entirely unseen during training and hyperparameter optimization; (4) Regularization optimization: LightGBM *L*_1_ and *L*_2_ regularization parameters are systematically optimized through Bayesian optimization using 100 Optuna trials, effectively balancing model complexity and generalization capacity.

## Results and discussion

### Hyperparameter optimization

The LightGBM classifier for Khib site prediction requires systematic hyperparameter optimization to achieve optimal predictive performance. This optimization was performed using Optuna ^69^, a state-of-the-art framework that employs Bayesian optimization with Tree-structured Parzen Estimators (TPE) to efficiently explore hyperparameter spaces. The optimization process was configured to maximize the AUC as the objective function, with a total of 100 trials executed to ensure a comprehensive exploration of the parameter space.

Optuna TPE algorithm iteratively refines hyperparameter selection through a probabilistic model that balances the exploration of unexplored regions with the exploitation of promising parameter combinations. This approach demonstrated convergence behaviour with optimal AUC values stabilizing after approximately 60–70 trials, indicating efficient parameter space exploration. Table 2 lists the eight main hyperparameters we focused on: number of estimators (n_estimators), learning rate, maximum tree depth, number of leaves, feature subsampling ratio (colsample_bytree), data subsampling ratio (subsample), and minimum child samples. The parameter ranges were chosen following common LightGBM best practices.

The hyperparameter optimization yielded an improvement over default LightGBM settings, with the optimized configuration achieving an AUC improvement of approximately 4.2% compared to the baseline model. Convergence analysis revealed that the optimization process efficiently identified near-optimal parameter combinations, with minimal performance variance observed in the final 20 trials, confirming the robustness of the selected hyperparameters.

**Table 2:** Key parameters of the LightGBM classifier and optimal settings.

Parameter	Default Value	Tested Range	Optimal Setting
No. of estimators	100	[100–1000]	500
Learning rate	0.1	[0.01–0.1]	0.1
Maximum depth	−1	[−1–12]	11
Number of leaves	31	[31–150]	89
*L*_1_ regularization (α)	0	$\:{[10}^{-8}-10]$	$\:1.15\times\:{10}^{-5}$
*L*_2_ regularization (γ)	0	$\:{[10}^{-8}-10]$	$\:4.4\times\:{10}^{-8}$

### Comparative evaluation of feature representation methods on validation datasets

The development of an effective predictor for Khib sites using LightGBM necessitates a comprehensive evaluation of various feature representation methods to determine their discriminative capacity. This investigation comprises five distinct protein-encoding schemes that capture complementary aspects of protein sequences. The embedding-based scheme, ESM-2, leverages deep learning to extract contextual information from protein sequences. In contrast, the descriptors-based schemes, C-CTD, T-CTD, and D-CTD, encode sequence-level features that represent compositional, transitional, and distributional characteristics, respectively. The property-based scheme, AAindex, encodes intrinsic physicochemical properties of amino acids, providing insights into their biochemical attributes.

To maximize the feature space and enhance predictive performance, these encoding schemes were systematically integrated into hybrid feature sets, resulting in four additional combinations as detailed in Tables S4–S6. These combinations include: (1) CTD, which integrates C-CTD, T-CTD, and D-CTD; (2) CTD + ESM; (3) AAindex + ESM; and (4) AAindex + CTD. Additionally, a comprehensive “All Features” set was created by integrating all five individual encoding methods. Performance assessment was conducted across three species-specific datasets (*H. sapiens*, *T. gondii*, and *O. sativa*) using stratified 10-fold cross-validation to ensure balanced class distributions. The performance metrics revealed distinct patterns across the datasets, with notable variations in the efficacy of different feature representation methods (Tables S4–S6).

In the *H. sapiens* dataset (Table S4), the AAindex encoding method demonstrated excellent performance among individual representations, achieving an ACC of 0.780 and an AUC of 0.857. This performance significantly surpassed those of other individual methods (ESM, C-CTD, T-CTD, and D-CTD) by margins of 10.5% −19.82% for ACC and 10.58% −21.56% for AUC. The integration of AAindex with other encoding methods yielded statistically significant improvements. Specifically, the AAindex + CTD combination achieved an ACC of 0.790 and an AUC of 0.872, while the comprehensive “All Features” approach demonstrated the highest overall performance with an ACC of 0.791 and an AUC of 0.874. Notably, the MCC, a balanced measure of classification quality, reached 0.584 with the “All Features” approach, indicating robust discriminative capacity.

Analysis of the *T. gondii* dataset (Table S5) revealed that the AAindex encoding scheme again exhibited advanced performance among individual methods, with ACC and AUC values of 0.741 and 0.816, respectively. These values exceeded the average ACC and AUC of the other four individual encoding methods by 6.77% and 6.38%, respectively. Hybrid feature combinations demonstrated marked improvements, with the AAindex + CTD combination achieving an ACC of 0.764 and an AUC of 0.848. The “All Features” integration yielded the highest performance metrics (ACC of 0.766, and AUC of 0.852), ensuring that a comprehensive feature representation captures complementary aspects of the sequence information, effectively for this organism.

For the *O. sativa* dataset (Table S6), the individual encoding methods demonstrated moderate performance, with AAindex again outperforming other individual methods (ACC of 0.735, and AUC of 0.802). The hybrid feature combinations showed incremental enhancements, with AAindex + CTD achieving an ACC of 0.755 and an AUC of 0.834. The “All Features” integration demonstrated the highest overall performance (ACC of 0.760, and AUC of 0.838), with a notable improvement in sensitivity (SN of 0.806) compared to individual methods. The MCC value of 0.522 for the “All Features” approach indicates notable improvement over individual encoding schemes, confirming the benefits of feature integration for this organism.

To validate the robustness of our approach and ensure that the observed patterns are not model-specific, parallel experiments were performed using Random Forest (RF), XGBoost, and CatBoost classifiers, with the *H. sapiens* dataset serving as a representative case. The supplementary material (Tables S7–S9) presents these comparative results in detail. Importantly, all classifiers exhibited consistent patterns wherein hybrid feature representations enhanced performance across all evaluation metrics. However, LightGBM consistently demonstrated better efficacy compared to the alternative classifiers, affirming its particular suitability for this prediction task.

**Fig. 3:**
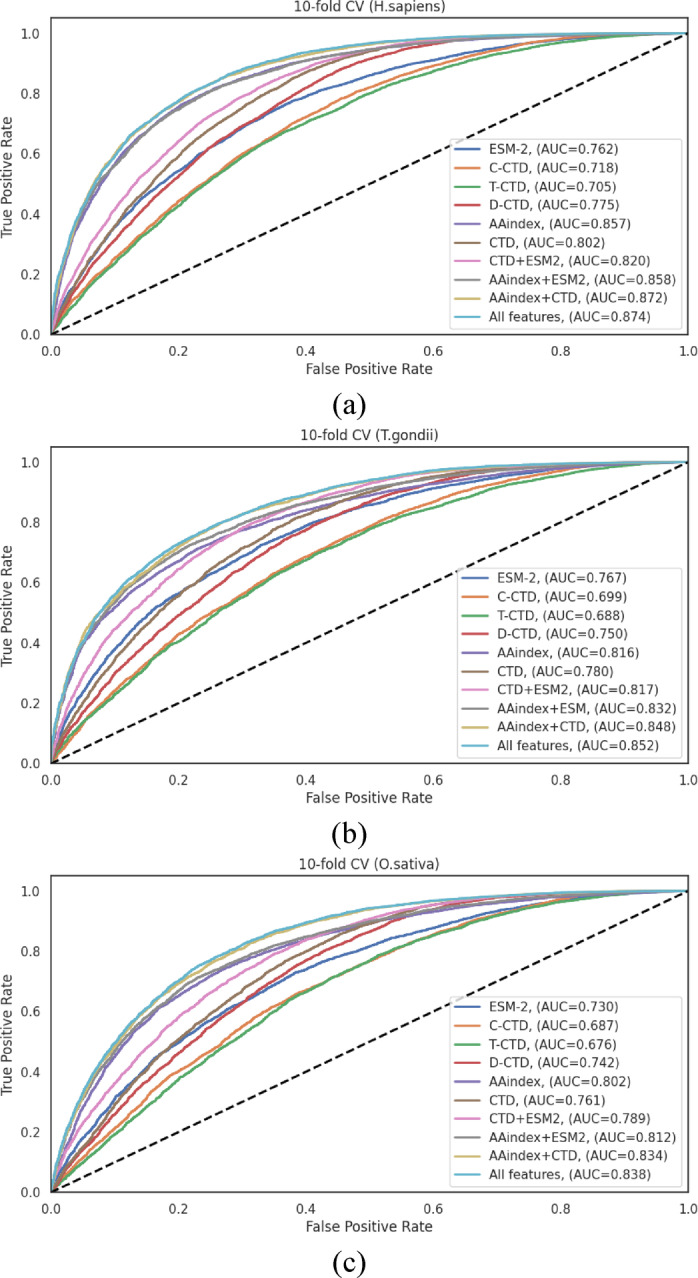
ROC curves of various feature representation methods on the 10-fold cross-validation data of (**a**) *H. sapiens*, (**b**) *T. gondii* and (**c**) *O. sativa* datasets.

The performance differentials across feature representation methods, visualized through ROC curves in Fig. 3, clearly illustrate the advantages of hybrid approaches. The consistent pattern observed across all three species, where integrated feature sets outperformed individual encoding schemes, strongly ensures that different encoding methods capture complementary aspects of the sequence information, and their integration provides a more comprehensive representation of the characteristics relevant to Khib site prediction.

### Comparative evaluation of feature representation methods on test sets

To evaluate the generalization ability of the LightGBM-based Khib prediction model, 10% of each species-specific dataset was reserved as an independent test set. The model performance on these sets, using various encoding schemes and their combinations, closely aligned with the cross-validation results (Sec."Comparative evaluation of feature representation methods on validation datasets"). Notably, AAindex encoding demonstrated stable performance across all species achieving AUC scores of 0.866 (H. sapiens), 0.828 (T. gondii), and 0.802 (O. sativa) highlighting its strong and consistent discriminative power for Khib site prediction based on physicochemical properties.

The incremental integration of complementary feature representations yielded a progressive enhancement in predictive performance across all datasets. The initial combination of CTD descriptors established a foundational improvement over individual encoding. This performance was subsequently enhanced through the sequential integration of ESM-2 and AAindex features, culminating in the comprehensive “All Features” approach that consistently demonstrated good performance across all evaluation metrics.

On the *H. sapiens* independent test set (Table 3), the comprehensive feature integration (“All Features”) achieved the highest performance metrics: ACC = 0.814, SN = 0.840, SP = 0.787, F1-score = 0.821, MCC = 0.629, and AUC = 0.890. Notably, the AAindex + CTD combination also demonstrated robust performance (ACC = 0.808, and AUC = 0.885), revealing that these feature types capture complementary aspects of the sequence information, particularly relevant to Khib site prediction in human proteins. The performance differential between the best individual encoding (AAindex with ACC = 0.793, and AUC = 0.866) and the optimal feature combination (“All Features”) represented a statistically significant improvement of 2.65% in accuracy and 2.77% in AUC, underscoring the value of feature integration.

For the *T. gondii* independent test set (Table 4), the “All Features” approach similarly demonstrated enhanced performance, achieving ACC = 0.781, SN = 0.799, SP = 0.763, F1-score = 0.781, MCC = 0.562, and AUC = 0.867. The performance enhancement from the best individual encoding (AAindex with ACC = 0.756, and AUC = 0.828) to the optimal feature combination represented an improvement of 3.31% in accuracy and 4.71% in AUC. The AAindex + ESM combination also performed well (ACC = 0.773, and AUC = 0.853), showing that the integration of physicochemical properties with evolutionary information provides valuable discriminative features for this organism.

Analysis of the *O. sativa* independent test set (Table 5) revealed that the “All Features” integration achieved the highest overall performance with ACC = 0.764, SN = 0.788, SP = 0.737, F1-score = 0.776, MCC = 0.526, and AUC = 0.835. Interestingly, the AAindex + CTD combination demonstrated comparable performance (ACC = 0.763, and AUC = 0.839). The performance improvement from the best individual encoding (AAindex with ACC = 0.728, and AUC = 0.802) to the optimal feature combination represented an enhancement of 4.95% in accuracy and 4.11% in AUC.

**Fig. 4:**
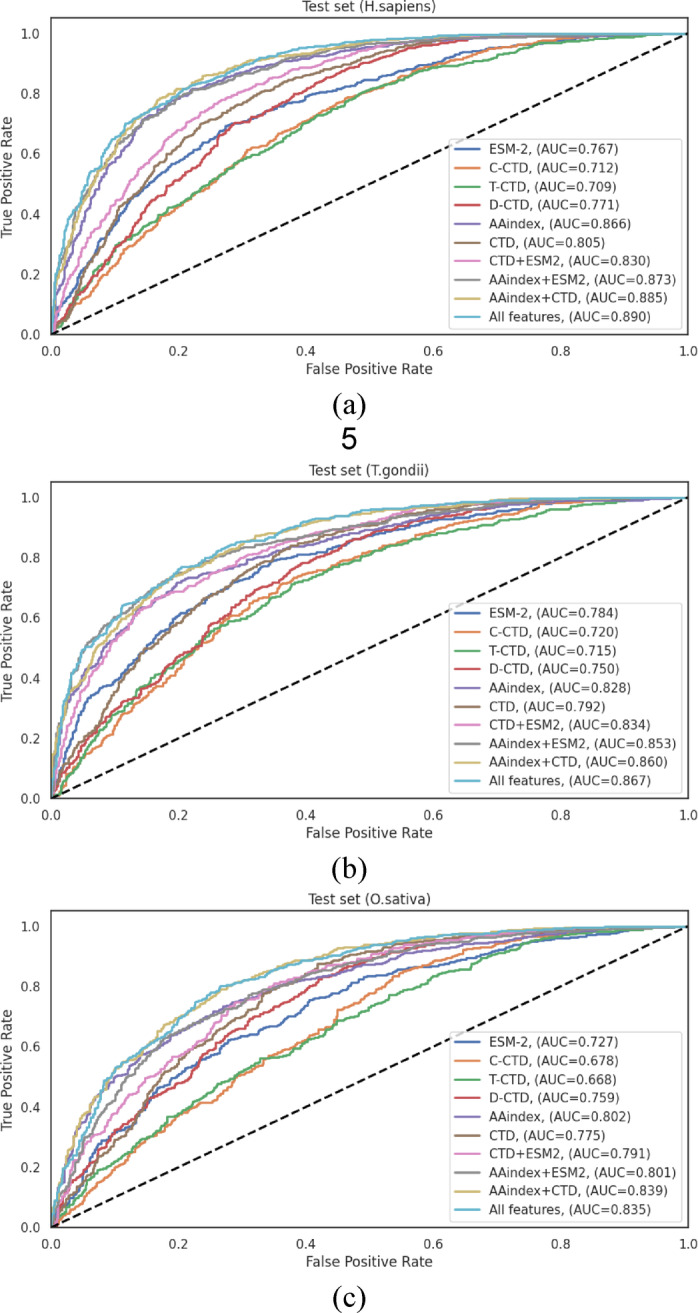
ROC curves of various feature representation methods on the independent test sets of (**a**) *H*. *sapiens*, (**b***) T. gondii* and (**c**) *O. sativa* datasets.

The ROC curves illustrated in Fig. 4 visually represent the performance differentials across the various feature representation methods on the independent test sets. These curves provide clear evidence for the progressive enhancement in discriminative capacity achieved through feature integration, with the “All Features” approach consistently demonstrating the largest AUC across all datasets.

The steady improvement in performance from combining features, seen in both cross-validation and independent tests, shows that different encoding methods capture useful and complementary information about protein sequences for predicting Khib sites. The integration of these complementary features provides a more comprehensive representation of the sequence characteristics, enabling more accurate and robust prediction of Khib sites across diverse organisms.

**Table 3:** LightGBM classification performance with different feature representation methods on the independent test set of the H. sapiens dataset. Boldface values indicate the best value for each metric.

Feature Representation	ACC	SN	SP	PR	F1	MCC	AUC
ESM	0.703	0.728	0.677	0.698	0.712	0.405	0.767
C-CTD	0.656	0.712	0.599	0.645	0.677	0.313	0.712
T-CTD	0.647	0.680	0.614	0.644	0.661	0.295	0.709
D-CTD	0.705	0.783	0.625	0.681	0.729	0.413	0.771
AAindex	0.793	0.800	0.786	0.793	0.787	0.586	0.866
CTD	0.738	0.808	0.666	0.713	0.757	0.479	0.806
CTD + ESM	0.754	0.822	0.684	0.727	0.772	0.511	0.830
AAindex + ESM	0.794	0.807	0.780	0.790	0.798	0.587	0.873
AAindex + CTD	0.808	0.839	0.777	0.794	0.816	0.617	0.885
All features	**0.814**	**0.840**	**0.787**	**0.802**	**0.821**	**0.629**	**0.890**

**Table 4:** LightGBM classification performance with different feature representation methods on the independent test set of the *T. gondii *dataset. Boldface values indicate the best performance for each metric.

Feature Representation	ACC	SN	SP	PR	F1	MCC	AUC
ESM	0.716	0.722	0.711	0706	0.714	0.433	0.784
C-CTD	0.674	0.731	0.620	0.649	0.687	0.352	0.720
T-CTD	0.656	0.674	0.640	0.643	0.658	0.313	0.715
D-CTD	0.689	0.763	0.618	0.658	0.707	0.385	0.750
AAindex	0.756	0.734	0.777	0.760	0.747	0.512	0.828
CTD	0.721	0.789	0.656	0.688	0.735	0.449	0.792
CTD+ESM	0.744	0.774	0.716	0.724	0.748	0.490	0.834
AAindex+ESM	0.773	0.760	0.786	0.773	0.767	0.546	0.853
AAindex+CTD	0.766	0.784	0.749	0.750	0.767	0.533	0.860
All features	**0.781**	**0.799**	**0.763**	**0.764**	**0.781**	**0.562**	**0.867**

**Table 5:** LightGBM classification performance with different feature representation methods on the independent test set of the *O. sativa* dataset. Boldface values indicate the best performance for each metric.

Feature Representation	ACC	SN	SP	PR	F1	MCC	AUC
ESM	0.663	0.660	0.667	0.681	0.670	0.326	0.727
C-CTD	0.618	0.674	0.557	0.622	0.647	0.233	0.678
T-CTD	0.617	0.677	0.552	0.620	0.647	0.231	0.668
D-CTD	0.693	0.766	0.615	0.682	0.722	0.386	0.759
AAindex	0.728	0.725	0.731	0.744	0.735	0.456	0.802
CTD	0.724	0.794	0.648	0.709	0.749	0.448	0.775
CTD+ESM	0.723	0.760	0.684	0.722	0.740	0.445	0.791
AAindex+ESM	0.723	0.715	0.732	0.742	0.728	0.447	0.801
AAindex+CTD	0.763	**0.803**	0.719	0.755	0.779	0.525	0.839
All features	**0.764**	0.788	**0.737**	**0.764**	**0.776**	**0.526**	**0.835**

### Performance analysis of feature selection algorithms

The HyLightKhib framework has a comprehensive two-phase feature engineering strategy to optimize predictive performance. The first phase focuses on integrating diverse feature representation techniques to capture complementary aspects of protein sequences. While this integration enhances discriminative capacity, it inherently introduces potential redundancy and noise that may impair computational efficiency and model generalization. Therefore, the second phase depends on feature selection algorithms to identify the most informative feature subset, with the primary objective of reducing computational overhead, while preserving model accuracy.

To identify the most effective dimensionality reduction approach, we systematically evaluated five established feature selection techniques: ANOVA, RFE, Lasso, Elastic Net, and MI. The evaluation was conducted across three species-specific datasets (*H. sapiens*, *T. gondii*, and *O. sativa*), with performance assessed through rigorous 10-fold cross-validation detailed in supplementary material Table S10, and independent test set validation Table S11.

**Fig. 5:**
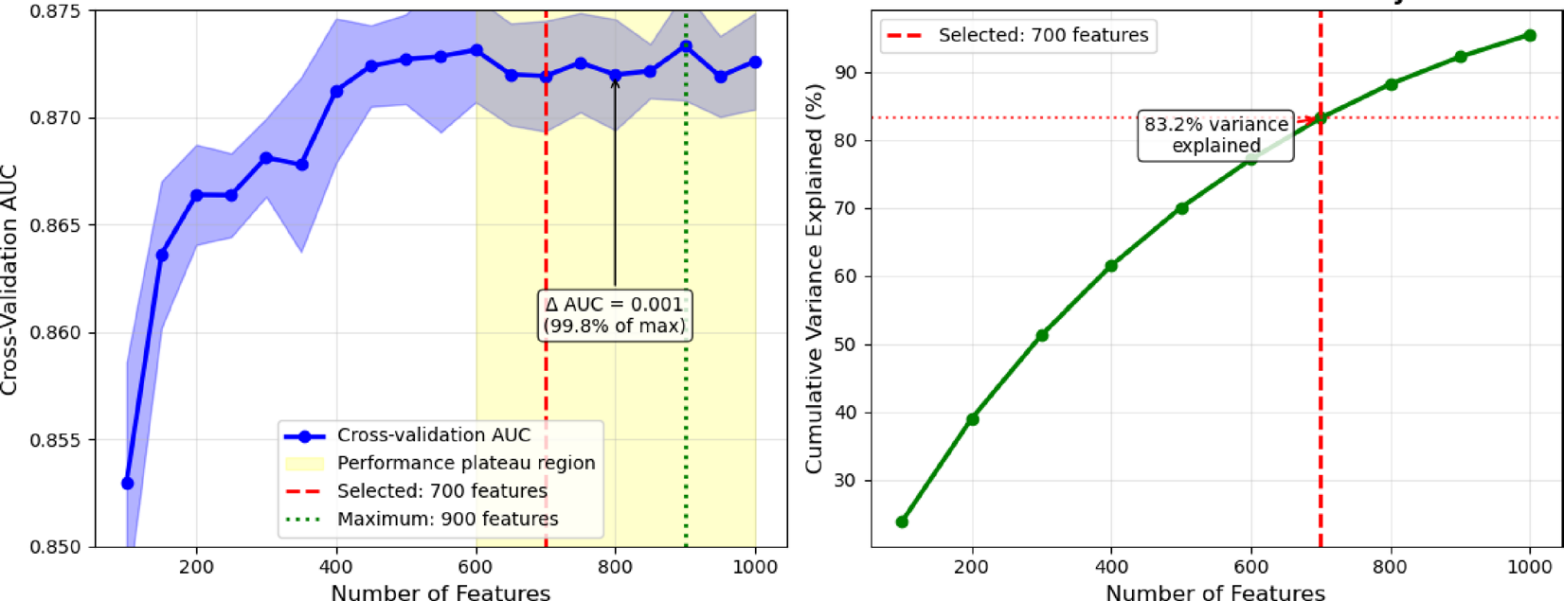
Systematic optimization for mutual-information-based feature selection threshold.

The original integrated feature set comprised 1,487 dimensions. Systematic evaluation across *H. sapiens* feature subsets from 100 to 1,000 features revealed that performance plateaus beyond 600 features, with cross-validation AUC values remaining stable (0.872–0.873) across the 600–1000 feature range (Fig. 5). While the maximum AUC (0.873) was achieved at 900 features, the performance difference from that obtained with 700 features was minimal (0.001 AUC units) and not statistically significant (*p* = 0.31). We selected 700 features as the optimal balance between performance and computational efficiency, as this threshold achieved 99.8% of the maximum performance level, while representing 83.2% of total mutual information variance. This leads to a reduction of the computational overhead by 22% compared to the case of 900 features.

As detailed in Table 6, MI-based feature selection achieved balanced dimensionality reduction across all three feature categories (ESM, CTD, and AAindex), but with species-specific variations in the proportional representation of each category. For the *H. sapiens* dataset, the original 1,487 dimensions were reduced to 700, with proportional contributions of ESM (34.14%), CTD (9.29%), and AAindex (56.57%). The *T. gondii* dataset exhibited a slightly different distribution, with ESM features constituting a larger proportion (40.14%) compared to *H. sapiens*. CTD features maintained a similar contribution (8.57%), and AAindex features represented a somewhat smaller proportion (51.29%). For the *O. sativa* dataset, the distribution more closely resembled that of *H. sapiens*, with ESM, CTD, and AAindex features contributing 33.86%, 10.29%, and 55.86%, respectively.

**Fig. 6:**
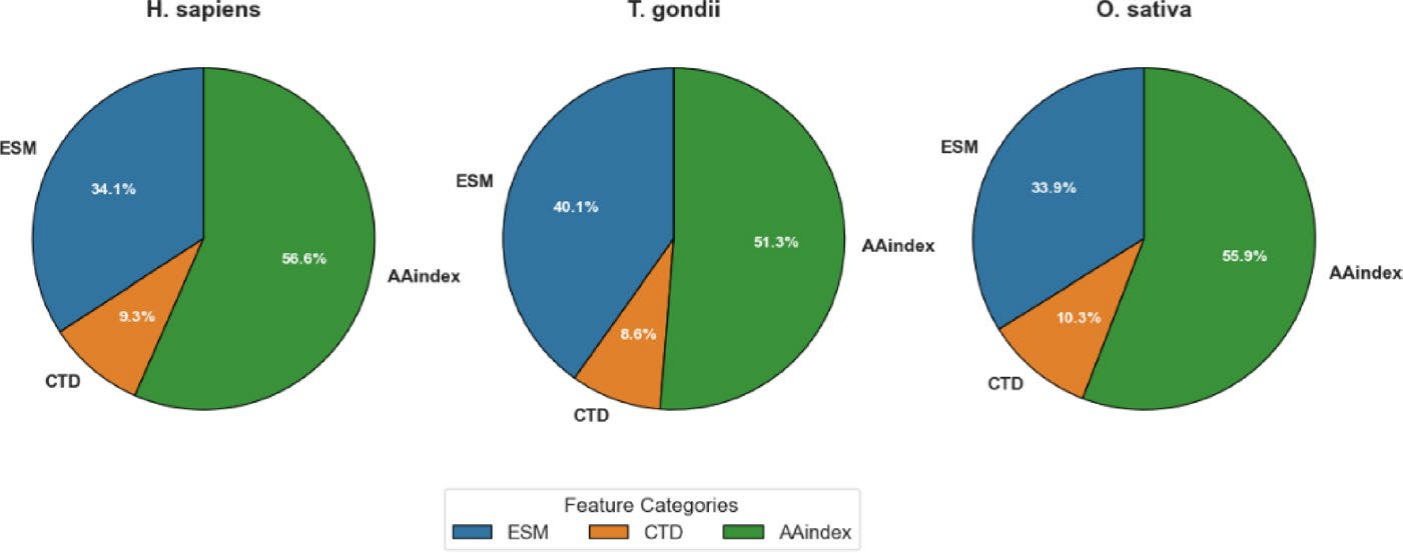
Visualization of feature category contribution after MI selection by dataset.

These differential distributions, visualized in Fig. 6, provide valuable insights into the relative importance of different feature categories across species. The fact that a large share of AAindex features (51.29–56.57%) was chosen in all datasets highlights how important physicochemical properties are for predicting Khib sites. This matches the strong results AAindex gave when used alone, as shown in Sec."Comparative evaluation of feature representation methods on validation datasets" and "Comparative evaluation of feature representation methods on test sets". The varying contribution of ESM features, particularly the higher proportion in *T. gondii* (40.14%) compared to those of *H. sapiens* (34.14%) and *O. sativa* (33.86%), reveals potential species-specific variations in the relevance of evolutionary information for Khib site prediction.

Elastic Net achieved more substantial dimensionality reductions through its inherent regularization mechanism, decreasing the feature dimensionality to 532, 446, and 447 for *H. sapiens*, *T. gondii*, and *O. sativa*, respectively. This more aggressive feature reduction did not consistently translate to advanced performance, highlighting the critical balance between dimensionality reduction and information preservation.

**Table 6:** Summary of the feature representation methods and their dimensionality before and after MI feature selection.

Dataset	Feature representation method	Original dimensions	Dimensions after MI selection
*H. sapiens*	ESM	480	239
	CTD	147	65
	AAindex	860	396
*T. gondii*	ESM	480	281
	CTD	147	60
	AAindex	860	359
*O. sativa*	ESM	480	237
	CTD	147	72
	AAindex	860	391
	All features	1487	700

The performance metrics of the various feature selection methods on the independent test sets are comprehensively presented in Table S11. These results reveal several significant patterns and insights regarding the efficacy of feature selection in enhancing model performance.

For the *H. sapiens* dataset, the original feature set established a robust baseline with an ACC of 0.814 and an AUC of 0.890. Among the feature selection methods, MI demonstrated effective performance, achieving comparable results with an ACC of 0.816 and an AUC of 0.893 while utilizing only 47% of the original features. ANOVA similarly achieved an AUC of 0.893, albeit with a slightly lower ACC of 0.801. The other methods maintained comparable performance levels, with Elastic Net showing modest improvement (ACC of 0.804 and AUC of 0.888) while achieving the most substantial dimensionality reduction. These results demonstrate that for the *H. sapiens* dataset, feature selection successfully maintains predictive performance while substantially reducing computational complexity.

Analysis of the *T. gondii* dataset revealed that the original feature set achieved an ACC of 0.781 and an AUC of 0.867. Both RFE and MI demonstrated enhanced discriminative capacity, with AUC values of 0.874 and 0.876, respectively.  The MI achieved notable performance metrics (ACC of 0.782, MCC of 0.551 and AUC of 0.876) over the original feature set while reducing dimensionality. Lasso demonstrated the most conservative performance among the methods evaluated (ACC of 0.759, and AUC of 0.851), revealing that its aggressive sparsity-inducing mechanism may eliminate some informative features for this particular organism. Elastic Net, with its balanced regularization approach, achieved performance metrics (ACC of 0.782, AUC of 0.866) comparable to those of the original feature set while requiring substantially fewer features.

For the *O. sativa* dataset, MI consistently achieved the highest performance among all evaluated methods, with an ACC of 0.765, an MCC of 0.517, and an AUC of 0.847. This performance represented an improvement over the original feature set (ACC of 0.764 and AUC of 0.835). The remaining feature selection methods demonstrated varying degrees of effectiveness, with ANOVA, RFE, and Elastic Net providing modest enhancements in specific metrics while maintaining an overall performance comparable to that of the original feature set. Lasso demonstrated the most substantial performance differential among the evaluated methods, achieving an ACC of 0.757 and an AUC of 0.829, ensuring potential species-specific variations in the effectiveness of sparsity-inducing feature selection.

**Fig. 7:**
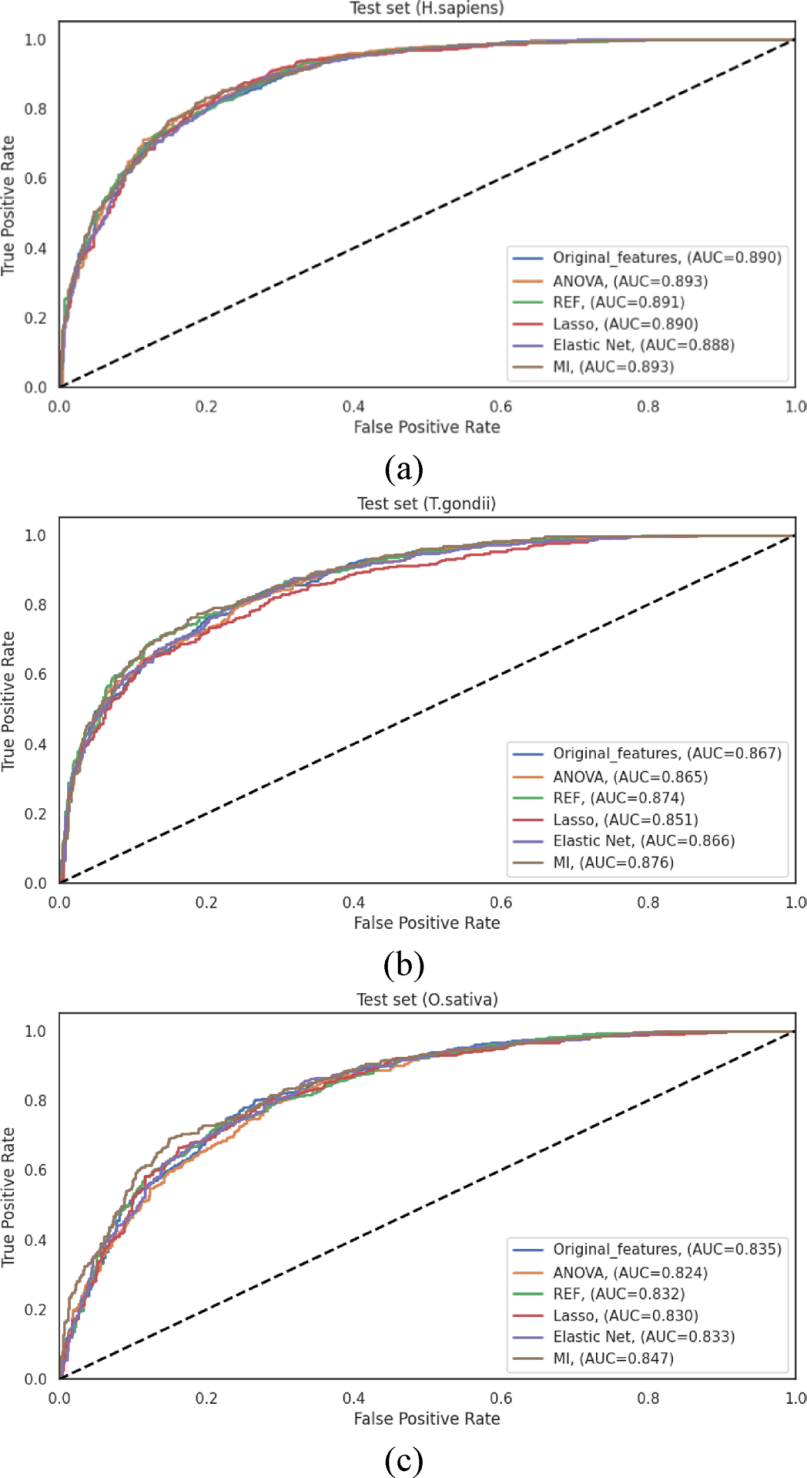
ROC curves illustrating the performance of LightGBM using different feature selection algorithms on the independent test sets of (**a**) *H. sapiens*, (**b**) *T. gondii*, and (**c**) *O. sativa* datasets.

The ROC curves illustrated in Fig. 7 represent the performance differentials across the various feature selection methods for all three species datasets. These curves corroborate the quantitative metrics presented in Table S11, demonstrating the consistently notable performance of MI-based feature selection across all datasets. The comprehensive evaluation reveals that while the original feature set provides robust baseline performance, feature selection methods successfully maintain or enhance predictive performance while substantially reducing computational complexity. MI-based feature selection achieved the highest performance metrics across all three datasets while reducing feature dimensionality by approximately 47%, effectively capturing nonlinear relationships between features and class labels.

### Performance evaluation of machine learning classifiers

Having established the optimal feature representation approach and identified MI-based feature selection as the most effective dimensionality reduction technique, we evaluated the performance of various machine learning algorithms for Khib site prediction. This comparative analysis encompassed seven widely-used classification algorithms: K-Nearest Neighbours (KNN) ^70^, Adaptive Boosting (AdaBoost) ^71^, Random Forest (RF) ^72^, Support Vector Machine (SVM) ^73^, XGBoost, CatBoost, and LightGBM. The evaluation was systematically conducted across the three species-specific datasets (*H. sapiens*, *T. gondii*, and *O. sativa*), utilizing the MI-selected feature subsets as input for each classifier.

To ensure a rigorous and fair comparison, each classifier hyperparameters were optimized through grid search cross-validation ^74^. The final configurations were as follows: KNN was employed with the Euclidean distance metric with *k* = 9 neighbors; RF was based on the Gini impurity criterion for node splitting with an ensemble of 500 decision trees; AdaBoost was implemented with a learning rate of 1.0 and 500 weak learners; SVM was employed with a polynomial kernel function; XGBoost was configured with a learning rate of 0.1, maximum tree depth of 15, 500 estimators, and both *L*_1_ and *L*_2_ regularization (λ = 1 and λ = 2, respectively); CatBoost was implemented with default hyperparameters and 500 iterations; and LightGBM was configured as detailed in Table 2. This systematic optimization strategy ensures that each algorithm performance was evaluated under optimal operating conditions.

**Fig. 8:**
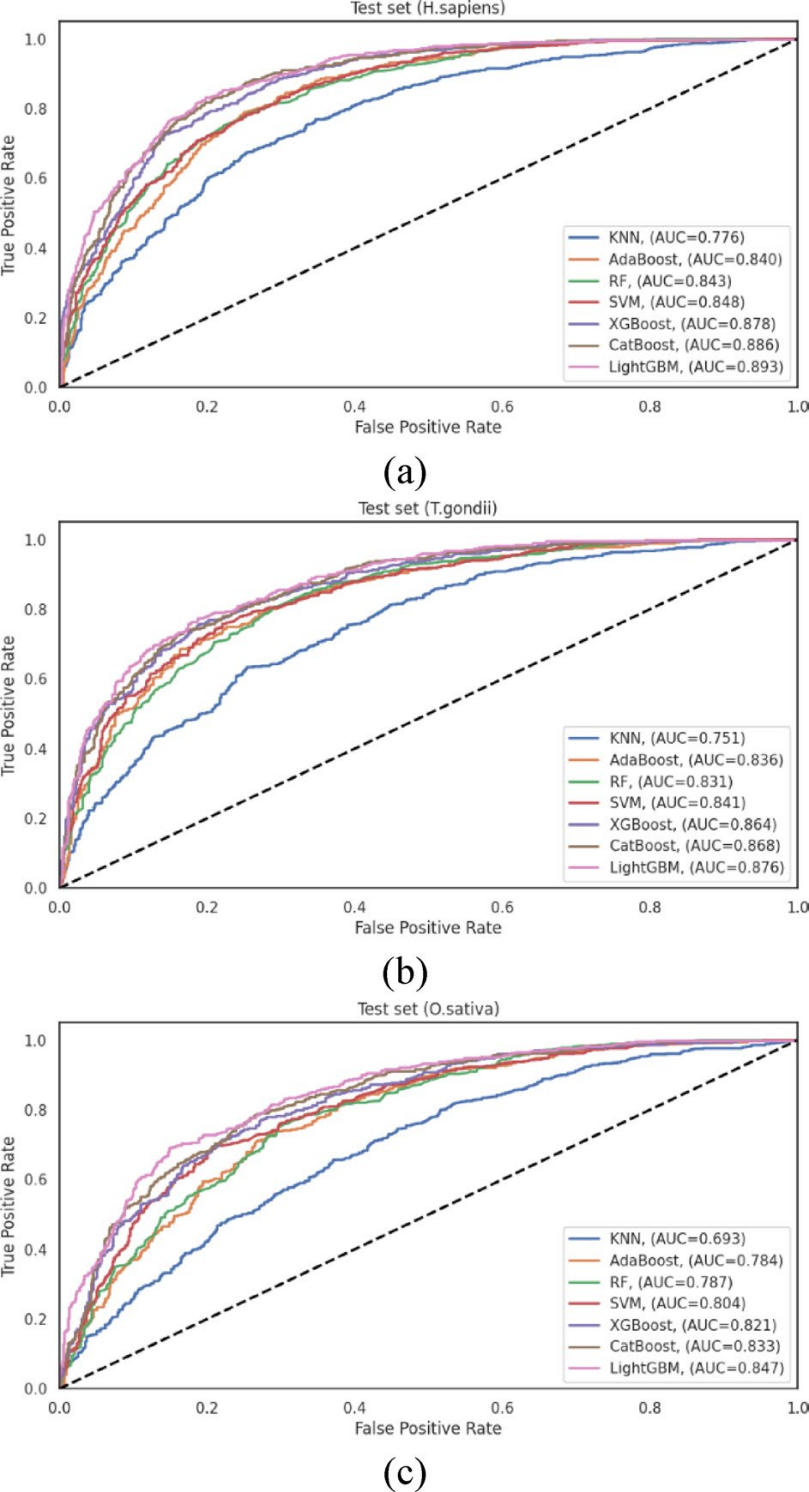
ROC curves showing the performance of different machine learning classifiers on the independent test sets of (**a**) *H. sapiens*, (**b**) *T. gondii* and (**c**) *O. sativa* datasets.

To ensure robust performance assessment, we implemented a dual validation strategy employing 10-fold cross-validation and independent test set evaluation. The cross-validation results are documented in Table S12. These results complement the independent test set evaluation presented in Table S13 and visualized in Fig. 8, offering a more comprehensive assessment of classifier performance across diverse data scenarios.

For the *H. sapiens* dataset, LightGBM achieved the highest performance among all evaluated classifiers, with an ACC = 0.816 and an AUC = 0.893. CatBoost followed closely with ACC = 0.807 and AUC = 0.886, and XGBoost performed competitively with ACC = 0.793 and AUC = 0.878. The traditional algorithms, SVM, RF, and AdaBoost, exhibited moderate performance, with AUC values of 0.848, 0.843, and 0.840, respectively. KNN demonstrated substantially lower discriminative capacity (AUC = 0.776), showing that distance-based classification approaches may be less suitable for this prediction task. The performance differential between LightGBM and KNN (AUC difference of 0.117) underscores the significant impact of algorithm selection on predictive efficacy for human Khib sites.

Analysis of the *T. gondii* dataset revealed similar performance patterns, with LightGBM achieving the highest overall metrics (ACC = 0.782 and AUC = 0.876). CatBoost (AUC = 0.868) and XGBoost (AUC = 0.864) demonstrated comparable performance, indicating that gradient-boosting algorithms generally excel at capturing the complex patterns associated with Khib sites in this organism. SVM maintained competitive performance with AUC = 0.841, while RF and AdaBoost exhibited moderate discriminative capacity with AUC = 0.831, and 0.836, respectively. As observed in the *H. sapiens* dataset, KNN achieved notably lower performance with AUC = 0.751, providing further evidence of the limitations of distance-based approaches for this prediction task.

The *O. sativa* dataset analysis reinforced the patterns observed in the other organisms, with LightGBM consistently achieving the highest performance among all classifiers with ACC = 0.765 and AUC = 0.847. CatBoost (AUC = 0.833) and XGBoost (AUC = 0.821) maintained their competitive performance, followed by SVM (AUC = 0.804). The ensemble-based approaches, RF and AdaBoost, demonstrated moderately lower performance with AUC = 0.787 and 0.784, respectively, while KNN exhibited the lowest discriminative capacity with AUC = 0.693. The substantial performance differential between LightGBM and KNN was most pronounced in this dataset, showing that the selection of an appropriate classification algorithm is particularly critical for plant Khib site prediction.

The consistent performance of LightGBM across all species datasets, validated by both cross-validation and independent testing, stems from its architectural strengths tailored to complex feature spaces like those in Khib site prediction. Its histogram-based binning enhances efficiency in handling high-dimensional data, while the leaf-wise tree growth with depth control captures complex nonlinear patterns without overfitting. Additionally, robust regularization contributes to its strong generalization ability. Although CatBoost and XGBoost also performed well, LightGBM consistently outperformed them, indicating that its specific design choices offer a distinct advantage for this classification task.

### Visualization of feature learning and cluster separation analysis

To evaluate HyLightKhib discriminative capacity, *t*-distributed stochastic neighbor embedding (*t*-SNE)^75^ was employed to visualize the feature transformation capabilities of the framework. The visualization analysis utilized the optimal configuration identified through a comprehensive performance evaluation, incorporating original input features with LightGBM classification following MI-based feature selection to maintain consistency with the best-performing model architecture.

**Fig. 9:**
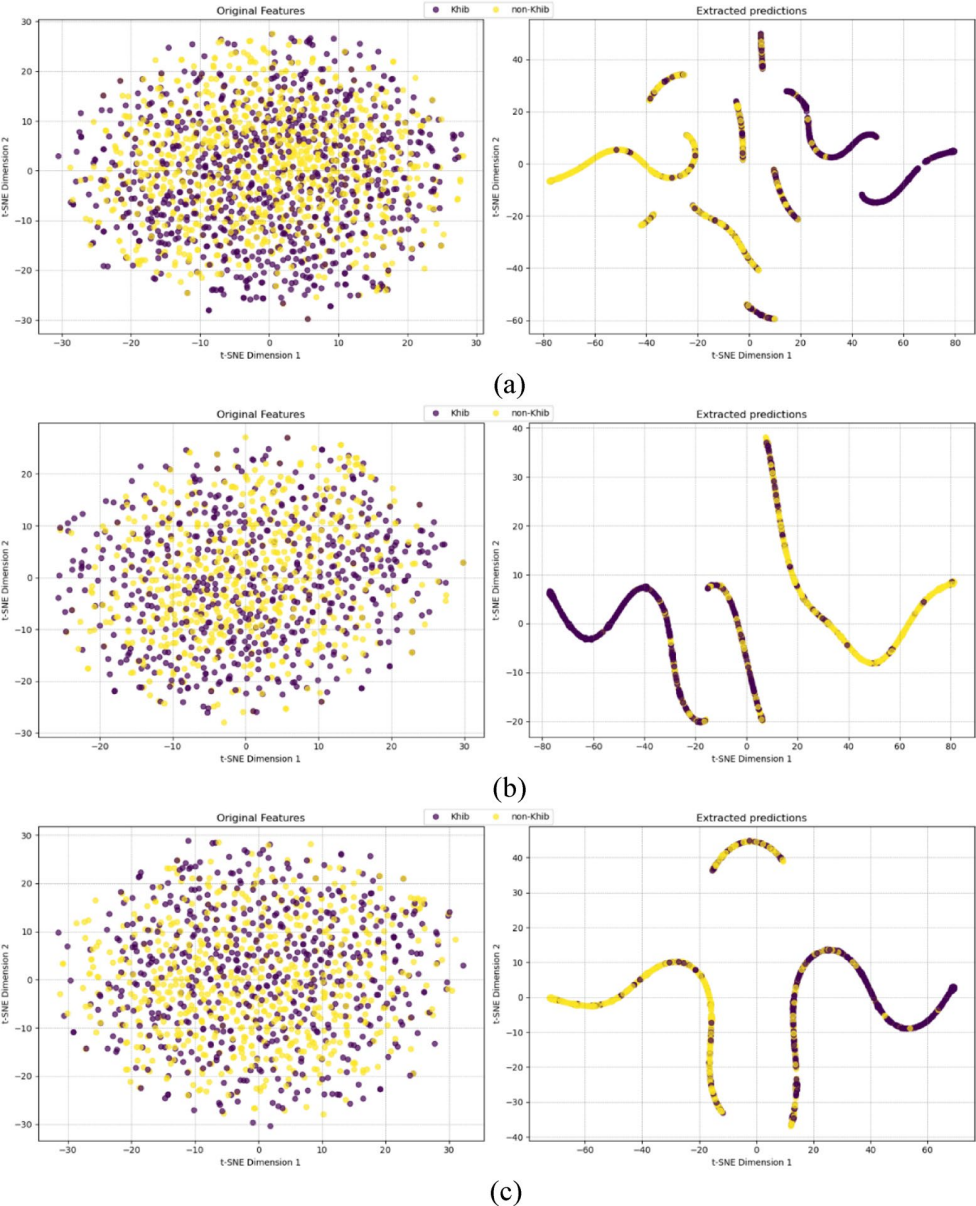
*t*-SNE visualization of input features (*left*) and extracted predictions (*right*) by HyLightKhib of (**a**) *H. sapiens*, (**b**) *T. gondii* and (**c**) *O. sativa* datasets.

The *t*-SNE visualizations for the species-specific *H. sapiens*, *T. gondii*, and *O. sativa* datasets shown in Fig. 9 demonstrate the effectiveness of our optimized framework in transforming the hybrid features into highly discriminative probability predictions. To provide a quantitative assessment beyond visual interpretation, silhouette scores and Davies-Bouldin indices were calculated for both the original feature space (left panels) and the LightGBM probability predictions (right panels).

The silhouette score measures how similar data points are to their cluster compared to other clusters, with values ranging from − 1 to 1, where higher values indicate better-defined clusters. Scores near 0 indicate overlapping clusters, while negative values indicate potential misclassification. The Davies-Bouldin index quantifies the average similarity between clusters, where lower values indicate better separation with more compact and well-separated clusters. Values closer to 0 represent optimal clustering quality.

For the *H. sapiens* dataset (Fig. 9a), the original feature space exhibited minimal class separation with a silhouette score of 0.008 and Davies-Bouldin index of 1.200, indicating substantial overlap between Khib-modified and non-modified lysine residues even after feature selection. The LightGBM probability predictions achieved substantial improvement with a silhouette score of 0.262 and the Davies-Bouldin index of 0.885. This improvement in silhouette score demonstrates the LightGBM ability to transform the original features into highly discriminative predictions.

The *T. gondii* dataset analysis (Fig. 9b) revealed the most pronounced discriminative transformation, with LightGBM probability predictions achieving a silhouette score of 0.285 and Davies-Bouldin index of 0.686 compared to those of the input features (silhouette score of 0.005, and Davies-Bouldin index of 1.100). This improvement in cluster separation metrics aligns with the 87.6% AUC performance reported in Sect. 3.5, demonstrating the LightGBM capacity to extract discriminative patterns from the optimally-selected feature subset for this organism.

For the *O. sativa* dataset (Fig. 9c), the LightGBM transformed the input features into probability predictions with a silhouette score of 0.205 and Davies-Bouldin index of 0.831, representing an improvement over the input feature space (silhouette score of 0.006, and Davies-Bouldin index of 1.163). This improvement confirms the LightGBM effectiveness in processing the input feature set for plant protein analysis.

### Comparative motif analysis and position-specific amino acid preferences

To further investigate the sequence characteristics underlying Khib site recognition and the varying classification performance across organisms, Two-Sample Logo analysis ^76^ was employed to identify position-specific amino acid preferences surrounding modification sites. The Two-Sample Logo is a statistical visualization tool that compares amino acid composition between two sequence sets (positive and negative samples) by calculating the difference in relative frequencies at each position and assessing statistical significance through hypothesis testing.

The Two-Sample Logo analysis was performed using a 43-residue window (−21 to + 21 positions relative to the central lysine) for all three species datasets. At each position, amino acid frequencies were compared between Khib-modified and non-modified sequences using a two-sample *t*-test with a significance threshold of *p*-value ≤ 0.05. The resulting logos display amino acids with statistically significant differences in their occurrence frequencies between the two sample groups.

Enriched amino acids represent residues that occur more frequently in Khib-modified sequences compared to non-modified sequences at specific positions, showing positive associations with modification propensity. Conversely, depleted amino acids represent residues that occur less frequently in Khib-modified sequences, indicating negative associations with modification likelihood. The height of each letter is proportional to the magnitude of the frequency difference, with larger letters indicating more statistically significant and biologically relevant associations.

**Fig. 10:**
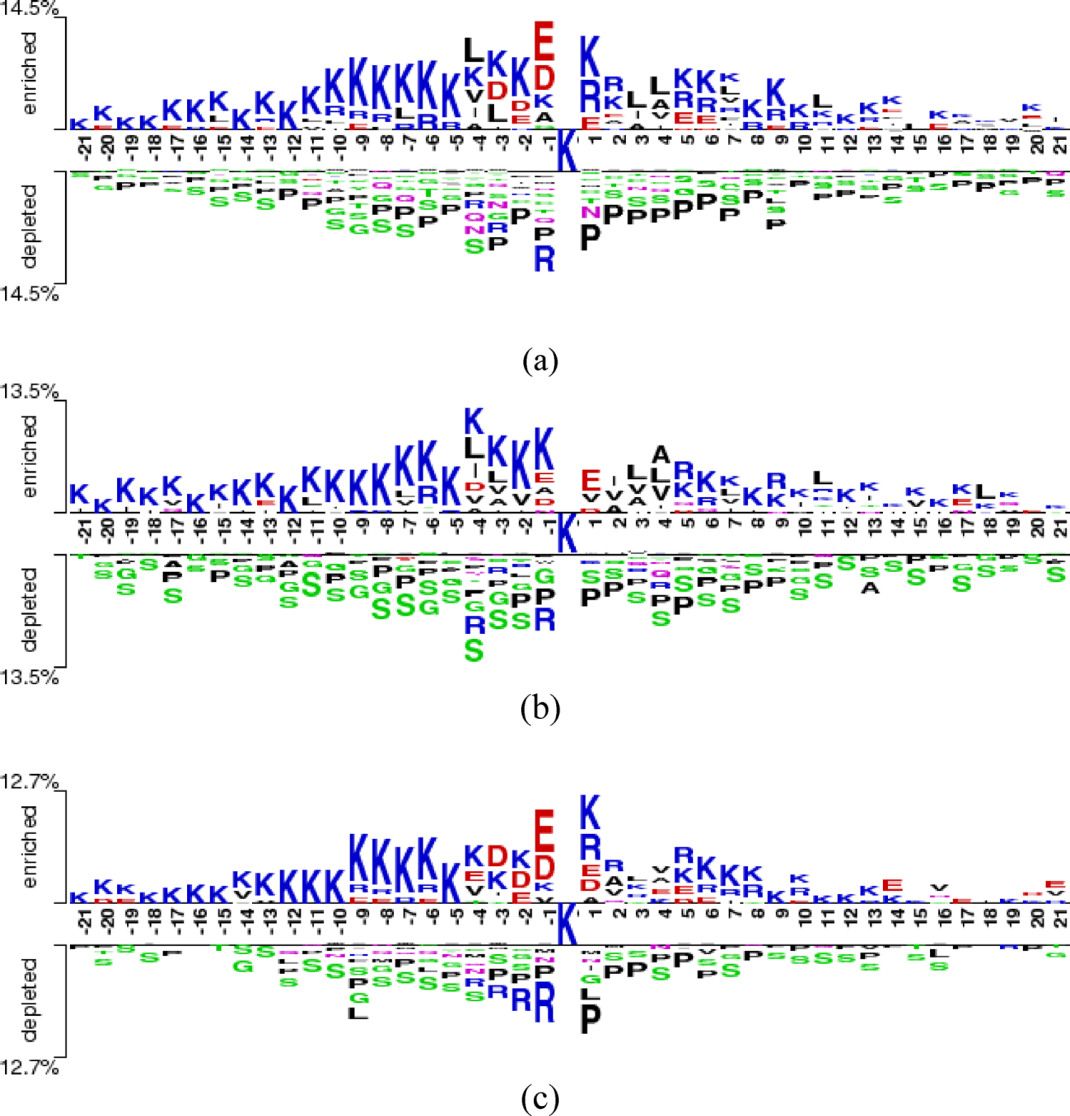
Comparison of the amino acid preferences near the Khib sites in the (**a**) *H. sapiens*, (**b**) *T. gondii* and (**c**) *O. sativa* datasets.

Two-sample logo analysis across three taxonomically diverse species reveals an evolutionary conservation of Khib recognition mechanisms alongside organism-specific adaptations. For *H. sapiens* (Fig. 10a), lysine (K) enrichment in upstream positions (−21 to −1) and glutamic acid (E) enrichment recommend cooperative recognition mechanisms within lysine-rich regions and favourable electrostatic environments for enzymatic machinery. Consistent proline (P) depletion indicates preferential modification in structured regions rather than helix-disrupted areas.

The *T. gondii* dataset (Fig. 10b) demonstrates conserved K enrichment patterns while exhibiting parasite-specific adaptations, including distinctive alanine (A) enrichment and reduced glutamic acid prominence compared to human sequences. Extensive serine (S) depletion (−8 to −10%) recommends spatial segregation mechanisms preventing phosphorylation-Khib crosstalk, while maintained proline depletion reinforces structured region preference.

Plant-specific adaptations in *O. sativa* (Fig. 10c) maintain core recognition features with pronounced E enrichment exceeding *T. gondii* levels, indicating enhanced acidic residue requirements in plant Khib systems. Unique arginine (R) depletion patterns (−6 to −8%) alongside consistent proline reduction recommend the avoidance of excessive positive charge density and structural disruptions.

Quantitative analysis reveals hierarchical motif signatures correlating directly with computational performance. Maximum lysine enrichment decreases across *H. sapiens* (14.5%), *T. gondii* (13.5%), and *O. sativa* (12.7%), corresponding precisely to classification performance with 89.3%, 87.6%, and 84.7% AUC, respectively.

###  Comparative analysis with state-of-the-art Khib prediction methods

To evaluate the performance of the HyLightKhib model in identifying Khib sites, its results were compared with those of existing Khib prediction tools: iLys-Khib, KhibPred, DeepKhib and ResNetKhib. iLys-Khib used a 35-residue window centred on the lysine and a fuzzy SVM. This approach reduces noise by giving different weights (fuzzy memberships) to samples depending on how relevant they are and how close they are to the class center. It incorporates three feature encoding methods: Amino Acid Factors (AAF), Binary Encoding (BE), and the Composition of *k*-spaced Amino Acid Pairs (CKSAAP) to capture the sequence context surrounding Khib sites. Feature selection used the maximum Relevance Minimum Redundancy (mRMR) method to retain the most informative features.

KhibPred used the same feature encoding as iLys-Khib but with a smaller window size of 29. It also used an ensemble SVM classifier to handle the imbalance in the dataset, where Khib sites are fewer than non-Khib sites. To handle this issue, the negative samples were divided into seven subsets, with an individual SVM trained on each subset, then combined with the positive samples. The ensemble model aggregated the predictions from all SVMs to make the final classification.

DeepKhib is a deep learning framework that employs a CNN architecture with a one-hot encoding approach. The model utilizes a four-layer architecture consisting of an input layer with one-hot encoding representation, a convolution layer containing four convolution sublayers (with 128 filters each of lengths 1, 3, 9, and 10) and two max pooling sublayers, a fully-connected layer incorporating global average pooling to prevent overfitting, and an output layer with sigmoid activation function for probability scoring.

ResNetKhib is the first cell-type-specific deep learning predictor for lysine Khib sites, employing a residual neural network (ResNet) architecture with a one-dimensional convolution and transfer learning strategy across different cell types and species. The model architecture consists of five key components: an input layer, an embedding layer, a convolution module containing six blocks with residual connections (first block with 64 filters, followed by five residual blocks), a fully-connected layer with 16 neurons for feature flattening, and an output layer with sigmoid activation for probability scoring. Both DeepKhib and ResNetKhib have 37-residue windows.

For a comprehensive methodological comparison, the SVM models documented in the iLys-Khib and KhibPred publications ^25,26^ and the CNN architectures described in DeepKhib and ResNetKhib ^6,28^ were reimplemented according to their original specifications. These models were subsequently trained and evaluated using our curated datasets under identical experimental conditions as our proposed method, thereby ensuring a fair and direct comparison. This approach eliminates potential confounding variables that might arise from differences in data preprocessing, partitioning strategies, or evaluation metrics.

Model training was performed using consistent cross-validation protocols across all compared methods, and performance was assessed on the same independent test sets to provide an unbiased evaluation of predictive capabilities. The comparative evaluation results across all benchmark methods are presented in Table S14.

As shown in Table S14, HyLightKhib achieved accuracy improvements of approximately 16.1%, 15.5%, 6.1%, and 2.9% over iLys-Khib, KhibPred, DeepKhib, and ResNetKhib, respectively, for the *H. sapiens* dataset. The improvements for the *T. gondii* dataset were 9.5%, 8.9%, 1.3%, and 0.6% compared to the same methods. For the *O. sativa* dataset, HyLightKhib achieved accuracy improvements of 13.8%, 11.35%, 8.7%, and 4.8% compared to the aforementioned predictors.

**Fig. 11:**
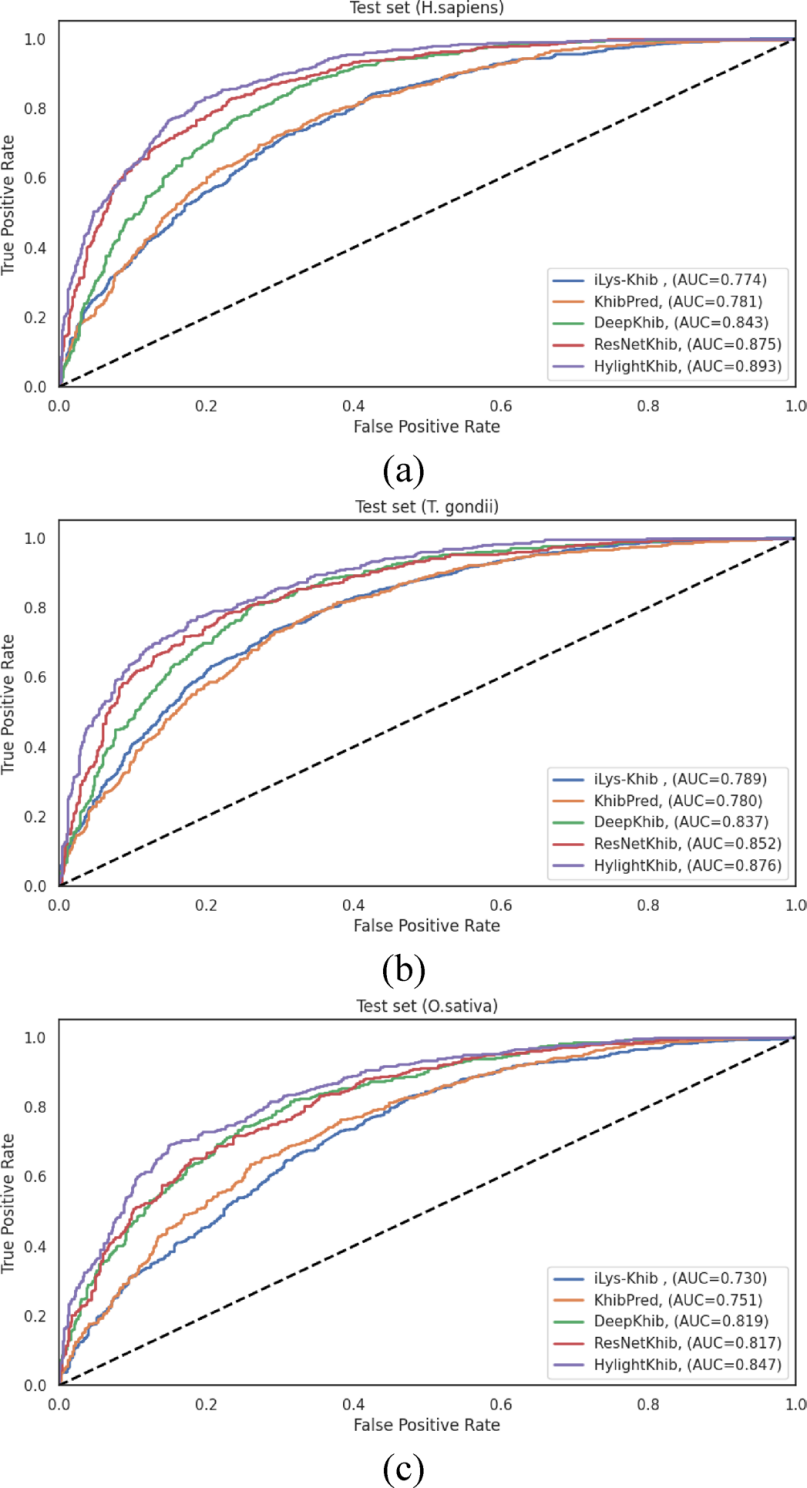
ROC curves comparing existing Khib predictors and HyLightKhib on the independent test sets for (**a**) *H. sapiens*, (**b**) *T. gondii*, and (**c**) *O. sativa* datasets.

The ROC curves and the corresponding AUC values presented in Fig. 11 reveal more modest improvements, particularly compared to recent deep learning methods. HyLightKhib achieved AUC values of 0.893, 0.876, and 0.847 for *H. sapiens*, *T. gondii*, and *O. sativa*, respectively. When compared to ResNetKhib, the most competitive baseline method, the AUC improvements were 1.8%, 2.4%, and 3.0% for the three datasets, respectively.

The statistical significance of AUC differences was assessed using the Hanley & McNeil method ^77^ for comparing correlated ROC curves derived from identical test sets. Although newer methods such as DeLong’s ^78^ test are now common, Hanley & McNeil remains a valid approach for estimating the significance of AUC differences in diagnostic and predictive modeling. Using this method, HyLightKhib demonstrated highly-significant improvements (*p* < 0.001) in overall existing predictors across the three species datasets, providing robust statistical evidence for its superior performance.

For the H. sapiens dataset, significant improvements were observed over ResNetKhib (*p* = 7.47 × 10⁻¹¹), DeepKhib (*p* < 10⁻¹⁶), iLys-Khib (*p* < 10⁻¹⁶), and KhibPred (*p* < 10⁻¹⁶). Similarly, the *T. gondii* dataset showed highly significant improvements over ResNetKhib (*p* = 6.11 × 10⁻¹⁰), DeepKhib (*p* < 10⁻¹⁶), iLys-Khib (*p* < 10⁻¹⁶), and KhibPred (*p* < 10⁻¹⁶). The *O. sativa* dataset exhibited the same pattern with significant improvements over ResNetKhib (*p* = 8.23 × 10⁻¹²), DeepKhib (*p* < 10⁻¹⁶), iLys-Khib (*p* < 10⁻¹⁶), and KhibPred (*p* < 10⁻¹⁶).

However, the primary advantage of HyLightKhib lies in its computational efficiency rather than dramatic performance gains. A comprehensive computational efficiency analysis was conducted to quantitatively assess the practical applicability of HyLightKhib compared to existing models. All experiments were performed on a standard desktop system (AMD Ryzen 5 7520U, 2.80 GHz, and 16.0 GB RAM). As detailed in Table 7, HyLightKhib demonstrates computational advantages across all evaluated metrics.

HyLightKhib demonstrated enhanced computational efficiency across all three datasets, with training times of 19.95, 19.36, and 21.77 seconds for the *H. sapiens*, *T. gondii*, and *O. sativa* datasets, respectively. When compared to existing methods, HyLightKhib achieved substantial training speedups: 92-166 times faster than DeepKhib (1994.11–3321.92 seconds), 19-27 times faster than ResNetKhib (414.59–548.05 seconds), 347-528 times faster than KhibPred (7236.3–10541.27 seconds), and 16–40 times faster than iLys-Khib (311.69–793.69 seconds). The most pronounced efficiency gains were observed against the ensemble-based KhibPred, highlighting HyLightKhib’s streamlined architecture.

Memory utilization analysis revealed HyLightKhib resource-efficient design, with peak consumption ranging from only 1.61–6.10 megabytes (MB) across all datasets. This represents substantial memory reductions compared to all baseline methods: 33-127 times less than DeepKhib (73.79-378.34 MB), 49-75 times less than ResNetKhib (121.07-298.23 MB), 42-109 times less than KhibPred (174.9-254.77 MB), and 10-121 times less than iLys-Khib (50.21–194.00 MB). The minimal memory footprint makes HyLightKhib particularly suitable for deployment in resource-constrained environments.

Inference performance evaluation demonstrated HyLightKhib capability for high-throughput applications, processing individual samples in 0.021–0.046 seconds across all datasets. This translates to significant inference accelerations: 63-132 times faster than DeepKhib (2.51–3.84 seconds), 24-48 times faster than ResNetKhib (1.014–1.29 seconds), 131-433 times faster than iLys-Khib (5.31–12.57 seconds), and remarkably 2139-4677 times faster than KhibPred (98.21–99.74 seconds). These performance metrics underscore HyLightKhib practical advantage for large-scale proteome-wide Khib site prediction tasks.

These quantitative computational benchmarks provide compelling empirical evidence for practical advantages in real-world applications. The significant reduction in computational resources, while maintaining competitive accuracy, makes HyLightKhib particularly suitable for large-scale proteomic analyses and integration into high-throughput experimental workflows. This empirical evaluation demonstrates that HyLightKhib offers an optimal balance between predictive performance and computational efficiency.

**Table 7:** Comparative analysis of computational efficiency metrics for Khib site prediction models across different dataset sizes.

Data	Method	Training time (sec)	Memory consumption (MB)	Inference speed/sample (sec)
*H. sapiens*	iLys-Khib	793.69	58.65	12.57
	Khibpred	10541.27	254.77	99.74
	DeepKhib	3321.92	378.34	3.84
	ResNetKhib	548.05	298.23	1.29
	HyLightKhib	19.95	6.10	0.029
*T. gondii*	iLys-Khib	311.69	194.00	5.31
	Khibpred	7236.3	174.9	98.21
	DeepKhib	2051.78	204.78	2.51
	ResNetKhib	496.70	121.07	1.014
	HyLightKhib	19.36	1.61	0.021
*O. sativa*	iLys-Khib	353.14	50.21	6.01
	Khibpred	7559.13	182.76	98.37
	DeepKhib	1994.11	73.79	2.88
	ResNetKhib	414.59	126.28	1.10
	HyLightKhib	21.77	2.25	0.046

## Conclusion

This study introduced HyLightKhib, a computational framework operating on 43-residue peptide sequences for predicting Khib sites by integrating ESM-2 embeddings, CTD descriptors, and selected amino acid physicochemical properties with mutual information-based feature selection and LightGBM classification. Comprehensive benchmarking across *human *, *parasite *, and *rice * proteomes revealed accuracy improvements ranging from 2.9 to 16.1% compared to existing tools, with more modest AUC improvements of 1.8-3.0% over the most competitive baseline methods. The primary contribution lies in computational efficiency, achieving 16-528 times faster training, 10-127 times lower memory consumption, and 24-4677 times higher inference speeds compared to deep learning alternatives, making the framework particularly suitable for high-throughput experimental workflows while maintaining competitive predictive accuracy.

Despite these advancements, several limitations must be acknowledged. The generalizability of the model to species beyond the three organisms tested remains uncertain. The current design does not incorporate three-dimensional structural information or consider potential crosstalk with other PTMs, both of which may influence site accessibility and biological function. Furthermore, the model was trained on an artificially balanced dataset, which may not reflect the true prevalence of Khib sites in vivo. Lastly, experimental validation of the predicted Khib sites remains essential to confirm their biological relevance and practical utility.

Looking ahead, future work should focus on extending the framework to additional species, integrating structural and multi-PTM information, and validating predictions through experimental assays. Nevertheless, HyLightKhib has several real-life applications in both biomedical and biotechnological research. It facilitates the investigation of epigenetic regulation and protein function by identifying candidate Khib sites, which are involved in key biological processes such as gene expression and chromatin remodeling. These insights can aid in the discovery of disease-associated biomarkers, guide experimental validation by prioritizing modification sites, and support therapeutic target identification. In addition, the framework can be applied to plant and microbial systems to enhance crop improvement and pathogen control, making it a valuable tool for advancing both healthcare and agricultural biotechnology. The framework computational scalability democratizes access to PTM prediction tools across research institutions with diverse resources, contributing to a deeper understanding of Khib modifications while promoting a balance between predictive accuracy and operational efficiency.

## Supplementary Information

Below is the link to the electronic supplementary material.

Supplementary Material 1

## Data Availability

All data generated or analysed during this study are included in this published article (and its Supplementary Information files).
