# Supplementary material for: An efficient machine-learning framework for predicting protein post-translational modification sites
Source: Sci Rep. 2025 Aug 25;15:31179. doi: 10.1038/s41598-025-13178-x (PMC12379237; doi:10.1038/s41598-025-13178-x)
Supplement: Supplementary file 1 — Supplementary Material 1 [file 41598_2025_13178_MOESM1_ESM.docx]

| **Table S1.** Performance comparison of various peptide window sizes on the Homo sapiens dataset. | | | | | | | | | | |
| --- | --- | --- | --- | --- | --- | --- | --- | --- | --- | --- |
| **Window size** | **10-fold cross validation** | | | |  | **Test set** | | | | |
|  | **ACC** | **F1** | **MCC** | **AUC** |  | **ACC** | **F1** | **MCC** | **AUC** |  |
| 47 | 0.789 | 0.796 | 0.579 | 0.872 |  | 0.807 | 0.814 | 0.615 | 0.888 |  |
| 45 | 0.790 | 0.798 | 0.582 | 0.873 |  | 0.816 | 0.823 | 0.633 | 0.893 |  |
| 43 | **0.792** | 0.800 | 0.586 | **0.874** |  | **0.816** | 0.825 | 0.633 | **0.898** |  |
| 41 | 0.786 | 0.794 | 0.574 | 0.871 |  | 0.816 | 0.824 | 0.634 | 0.895 |  |
| 39 | 0.791 | 0.799 | 0.585 | 0.872 |  | 0.824 | 0.832 | 0.649 | 0.894 |  |
| 37 | 0.791 | 0.798 | 0.584 | 0.874 |  | 0.814 | 0.821 | 0.629 | 0.893 |  |
| 35 | 0.791 | 0.801 | 0.585 | 0.873 |  | 0.814 | 0.823 | 0.629 | 0.886 |  |

| **Table S2.** Comparison of LightGBM performance using alternative feature representation methods on the test set. | | | | | | | |
| --- | --- | --- | --- | --- | --- | --- | --- |
| **Dataset** | **Feature selection** | **ACC** | **SN** | **PR** | **F1** | **MCC** | **AUC** |
| *H. sapiens* | ProtBERT | 0.643 | 0.660 | 0.647 | 0.654 | 0.285 | 0.705 |
|  | Word2vec | 0.688 | 0.698 | 0.693 | 0.695 | 0.376 | 0.748 |
|  | Onehot | 0.693 | 0.699 | 0.70 | 0.699 | 0.386 | 0.770 |
|  | Blosum62 | 0.731 | 0.749 | 0.730 | 0.739 | 0.461 | 0.792 |
| *T. gondii* | ProtBERT | 0.659 | 0.696 | 0.648 | 0.671 | 0.318 | 0.726 |
|  | Word2vec | 0.672 | 0.684 | 0.669 | 0.676 | 0.344 | 0.743 |
|  | Onehot | 0.682 | 0.711 | 0.673 | 0.692 | 0.365 | 0.744 |
|  | Blosum62 | 0.703 | 0.737 | 0.690 | 0.713 | 0.406 | 0.766 |
| *O. sativa* | ProtBERT | 0.625 | 0.648 | 0.629 | 0.638 | 0.250 | 0.671 |
|  | Word2vec | 0.648 | 0.689 | 0.645 | 0.667 | 0.296 | 0.689 |
|  | Onehot | 0.642 | 0.658 | 0.647 | 0.652 | 0.283 | 0.687 |
|  | Blosum62 | 0.649 | 0.707 | 0.642 | 0.673 | 0.298 | 0.708 |

| 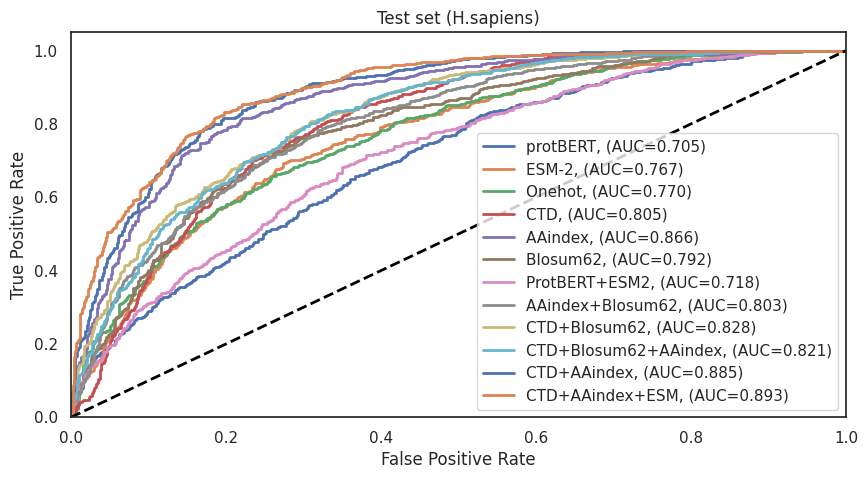 |
| --- |
| (a) |
| 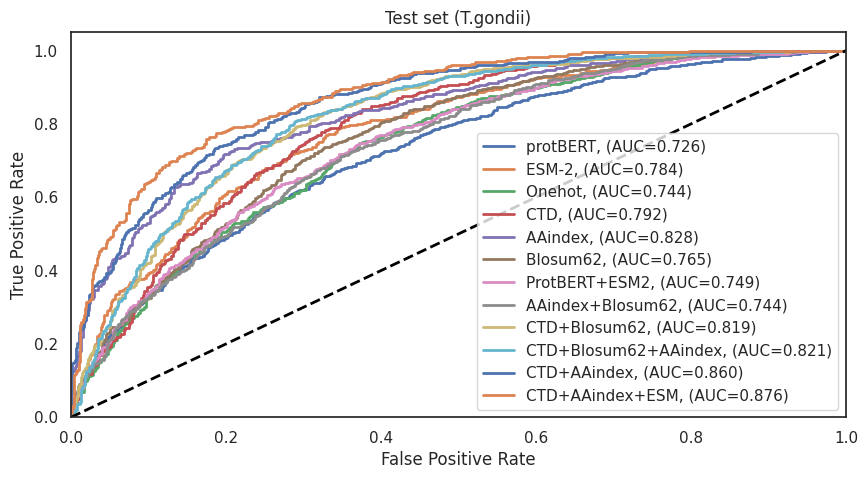 |
| (b) |
| 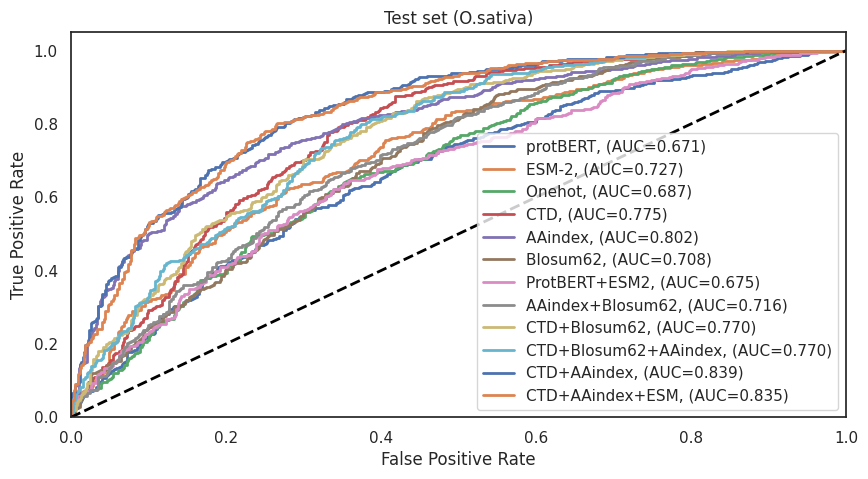 |
| (c) |
| **Fig. S1.** ROC curves for all examined feature representation methods before selecting the optimal set of representation methods, evaluated on the test sets of (a) H. sapiens, (b) T. gondii, and (c) O. sativa datasets. |

| **Table S3.** List of amino acid indices utilized in the study, along with their corresponding descriptions | |
| --- | --- |
| **AAindex** | **Description** |
| ARGP820101 | Hydrophobicity index |
| BIGC670101 | Residue volume |
| BROC820101 | Retention coefficient in TFA |
| DAYM780101 | Amino acid composition |
| EISD840101 | Consensus normalized hydrophobicity scale |
| FAUJ830101 | Hydrophobic parameter pi |
| FAUJ880108 | Localized electrical effect |
| FASG760102 | Melting point |
| FASG760101 | Molecular weight |
| GRAR740102 | Polarity |
| HARY940101 | Mean volumes of residues buried in protein interiors |
| HUTJ700103 | Entropy of formation |
| JUKT750101 | Amino acid distribution |
| KLEP840101 | Net charge |
| KRIW790103 | Side chain volume |
| KRIW790102 | Fraction of site occupied by water |
| MAXF760101 | Normalized frequency of alpha-helix |
| NAGK730102 | Normalized frequency of bata-structure |
| ROSM880102 | Side chain hydropathy |
| VELV850101 | Electron-ion interaction potential |

| **Table S4.** Performance of LightGBM classification using various feature representation methods on the 10-fold cross-validation of *H. sapiens* dataset*.* | | | | | | | |
| --- | --- | --- | --- | --- | --- | --- | --- |
| **Feature Representation** | **ACC** | **SN** | **SP** | **PR** | **F1** | **MCC** | **AUC** |
| ESM-2 | 0.694 | 0.715 | 0.674 | 0.686 | 0.700 | 0.389 | 0.762 |
| C-CTD | 0.661 | 0.719 | 0.604 | 0.644 | 0.679 | 0.325 | 0.718 |
| T-CTD | 0.651 | 0.696 | 0.607 | 0.639 | 0.666 | 0.304 | 0.705 |
| D-CTD | 0.706 | 0.792 | 0.621 | 0.676 | 0.729 | 0.419 | 0.775 |
| AAindex | 0.780 | 0.793 | 0.766 | 0.772 | 0.782 | 0.560 | 0.857 |
| CTD | 0.732 | 0.810 | 0.655 | 0.700 | 0.751 | 0.470 | 0.802 |
| CTD+ESM2 | 0.745 | 0.809 | 0.681 | 0.717 | 0.760 | 0.494 | 0.820 |
| AAindex+ESM2 | 0.778 | 0.791 | 0.765 | 0.770 | 0.781 | 0.556 | 0.858 |
| AAindex+CTD | 0.790 | 0.831 | 0.751 | 0.769 | 0.799 | 0.583 | 0.872 |
| All features | **0.791** | **0.829** | **0.754** | **0.770** | **0.798** | **0.584** | **0.874** |

| **Table S5.** Performance of LightGBM classification using various feature representation methods on the 10-fold cross-validation of *T.gondii* dataset*.* | | | | | | | |
| --- | --- | --- | --- | --- | --- | --- | --- |
| **Feature Representation** | **ACC** | **SN** | **SP** | **PR** | **F1** | **MCC** | **AUC** |
| ESM-2 | 0.694 | 0.706 | 0.682 | 0.690 | 0.698 | 0.388 | 0.767 |
| C-CTD | 0.642 | 0.688 | 0.596 | 0.631 | 0.658 | 0.285 | 0.699 |
| T-CTD | 0.638 | 0.668 | 0.608 | 0.631 | 0.649 | 0.276 | 0.688 |
| D-CTD | 0.688 | 0.758 | 0.617 | 0.665 | 0.709 | 0.379 | 0.750 |
| AAindex | 0.741 | 0.713 | 0.769 | 0.756 | 0.734 | 0.482 | 0.816 |
| CTD | 0.713 | 0.777 | 0.649 | 0.690 | 0.731 | 0.429 | 0.780 |
| CTD+ESM2 | 0.741 | 0.784 | 0.698 | 0.723 | 0.752 | 0.484 | 0.817 |
| AAindex+ESM2 | 0.752 | 0.741 | 0.763 | 0.759 | 0.750 | 0.504 | 0.832 |
| AAindex+CTD | 0.764 | 0.784 | 0.745 | 0.755 | 0.769 | 0.529 | 0.848 |
| All features | **0.766** | **0.784** | **0.748** | **0.758** | **0.771** | **0.533** | **0.852** |

| **Table S6.** Performance of LightGBM classification using various feature representation methods on the 10-fold cross-validation of *O.sativa* dataset. | | | | | | | |
| --- | --- | --- | --- | --- | --- | --- | --- |
| **Feature Representation** | **ACC** | **SN** | **SP** | **PR** | **F1** | **MCC** | **AUC** |
| ESM-2 | 0.669 | 0.676 | 0.661 | 0.664 | 0.679 | 0.337 | 0.730 |
| C-CTD | 0.634 | 0.683 | 0.586 | 0.621 | 0.650 | 0.270 | 0.687 |
| T-CTD | 0.632 | 0.674 | 0.591 | 0.620 | 0.646 | 0.266 | 0.676 |
| D-CTD | 0.684 | 0.770 | 0.599 | 0.656 | 0.708 | 0.375 | 0.742 |
| AAindex | 0.735 | 0.733 | 0.736 | 0.734 | 0.733 | 0.469 | 0.802 |
| CTD | 0.699 | 0.777 | 0.622 | 0.671 | 0.720 | 0.403 | 0.761 |
| CTD+ESM | 0.718 | 0.777 | 0.660 | 0.694 | 0.733 | 0.440 | 0.789 |
| AAindex+ESM | 0.739 | 0.740 | 0.739 | 0.738 | 0.739 | 0.478 | 0.812 |
| AAindex+CTD | 0.755 | 0.805 | 0.705 | 0.730 | 0.766 | 0.513 | 0.834 |
| All features | **0.760** | **0.806** | **0.714** | **0.737** | **0.770** | **0.522** | **0.838** |

| **Table S7**. Performance of Random Forest (RF) classification using various feature representation methods on the *H. sapiens* dataset. | | | | | | | | | | | | | | | |
| --- | --- | --- | --- | --- | --- | --- | --- | --- | --- | --- | --- | --- | --- | --- | --- |
| **Feature representation** | **10-fold Cross Validation set** | | | | | | |  | **Test set** | | | | | | |
|  | **ACC** | **SN** | **SP** | **PR** | **F1** | **MCC** | **AUC** |  | **ACC** | **SN** | **SP** | **PR** | **F1** | **MCC** | **AUC** |
| **ESM** | 0.687 | 0.688 | 0.686 | 0.686 | 0.687 | 0.374 | 0.749 |  | 0.687 | 0.673 | 0.702 | 0.699 | 0.687 | 0.375 | 0.755 |
| **C-CTD** | 0.668 | 0.714 | 0.622 | 0.653 | 0.682 | 0.337 | 0.723 |  | 0.664 | 0.705 | 0.622 | 0.657 | 0.680 | 0.329 | 0.720 |
| **T-CTD** | 0.655 | 0.697 | 0.614 | 0.643 | 0.669 | 0.312 | 0.709 |  | 0.644 | 0.659 | 0.628 | 0.645 | 0.652 | 0.287 | 0.713 |
| **D-CTD** | 0.693 | 0.812 | 0.575 | 0.655 | 0.725 | 0.397 | 0.749 |  | 0.685 | 0.778 | 0.588 | 0.659 | 0.714 | 0.374 | 0.747 |
| **AAindex** | 0.753 | 0.775 | 0.731 | 0.742 | 0.758 | 0.506 | 0.829 |  | 0.753 | 0.770 | 0.734 | 0.748 | 0.759 | 0.505 | 0.823 |
| **CTD** | 0.709 | 0.786 | 0.632 | 0.681 | 0.730 | 0.423 | 0.772 |  | 0.715 | 0.781 | 0.647 | 0.694 | 0.735 | 0.432 | 0.778 |
| **CTD+ESM** | 0.712 | 0.739 | 0.685 | 0.700 | 0.719 | 0.424 | 0.781 |  | 0.715 | 0.734 | 0.697 | 0.712 | 0.723 | 0.431 | 0.785 |
| **AAindex+ESM** | 0.715 | 0.717 | 0.712 | 0.713 | 0.715 | 0.429 | 0.792 |  | 0.740 | 0.733 | 0.748 | 0.749 | 0.741 | 0.481 | 0.804 |
| **AAindex+CTD** | 0.753 | 0.801 | 0.694 | 0.731 | 0.764 | 0.509 | 0.820 |  | 0.759 | 0.801 | 0.710 | 0.743 | 0.771 | 0.519 | 0.840 |
| **All features** | 0.751 | 0.808 | 0.693 | 0.724 | 0.764 | 0.505 | 0.828 |  | 0.762 | 0.813 | 0.709 | 0.741 | 0.776 | 0.525 | 0.843 |

| **Table S8**. Performance of XGBoost classification using various feature representation methods on the *H. sapiens* dataset. | | | | | | | | | | | | | | | |
| --- | --- | --- | --- | --- | --- | --- | --- | --- | --- | --- | --- | --- | --- | --- | --- |
| **Feature representation** | **10-fold Cross Validation set** | | | | | | |  | **Test set** | | | | | | |
|  | **ACC** | **SN** | **SP** | **PR** | **F1** | **MCC** | **AUC** |  | **ACC** | **SN** | **SP** | **PR** | **F1** | **MCC** | **AUC** |
| **ESM** | 0.691 | 0.711 | 0.672 | 0.684 | 0.697 | 0.383 | 0.738 |  | 0.709 | 0.725 | 0.694 | 0.708 | 0.716 | 0.419 | 0.768 |
| **C-CTD** | 0.665 | 0.729 | 0.599 | 0.651 | 0.688 | 0.331 | 0.719 |  | 0.668 | 0.730 | 0.606 | 0.649 | 0.687 | 0.339 | 0.724 |
| **T-CTD** | 0.649 | 0.694 | 0.604 | 0.636 | 0.664 | 0.299 | 0.702 |  | 0.642 | 0.679 | 0.602 | 0.637 | 0.657 | 0.283 | 0.708 |
| **D-CTD** | 0.706 | 0.813 | 0.600 | 0.669 | 0.734 | 0.422 | 0.774 |  | 0.700 | 0.801 | 0.597 | 0.671 | 0.730 | 0.407 | 0.766 |
| **AAindex** | 0.772 | 0.787 | 0.757 | 0.763 | 0.775 | 0.543 | 0.852 |  | 0.789 | 0.789 | 0.788 | 0.793 | 0.791 | 0.578 | 0.862 |
| **CTD** | 0.734 | 0.826 | 0.643 | 0.698 | 0.756 | 0.477 | 0.802 |  | 0.734 | 0.809 | 0.657 | 0.707 | 0.755 | 0.472 | 0.807 |
| **CTD+ESM** | 0.746 | 0.819 | 0.672 | 0.712 | 0.760 | 0.492 | 0.817 |  | 0.746 | 0.819 | 0.672 | 0.719 | 0.765 | 0.496 | 0.828 |
| **AAindex+ESM** | 0.761 | 0.779 | 0.743 | 0.751 | 0.765 | 0.522 | 0.843 |  | 0.769 | 0.791 | 0.746 | 0.761 | 0.776 | 0.538 | 0.857 |
| **AAindex+CTD** | 0.780 | 0.835 | 0.726 | 0.752 | 0.791 | 0.563 | 0.862 |  | 0.791 | 0.834 | 0.748 | 0.773 | 0.804 | 0.588 | 0.878 |
| **All features** | 0.780 | 0.832 | 0.728 | 0.753 | 0.791 | 0.563 | 0.862 |  | 0.793 | 0.837 | 0.748 | 0.773 | 0.804 | 0.588 | 0.878 |

| **Table S9**. Performance of CatBoost classification using various feature representation methods on the *H. sapiens* dataset. | | | | | | | | | | | | | | | |
| --- | --- | --- | --- | --- | --- | --- | --- | --- | --- | --- | --- | --- | --- | --- | --- |
| **Feature representation** | **10-fold Cross Validation set** | | | | | | |  | **Test set** | | | | | | |
|  | **ACC** | **SN** | **SP** | **PR** | **F1** | **MCC** | **AUC** |  | **ACC** | **SN** | **SP** | **PR** | **F1** | **MCC** | **AUC** |
| **ESM** | 0.701 | 0.725 | 0.677 | 0.691 | 0.708 | 0.403 | 0.765 |  | 0.705 | 0.723 | 0.687 | 0.703 | 0.713 | 0.411 | 0.770 |
| **C-CTD** | 0.679 | 0.752 | 0.607 | 0.658 | 0.700 | 0.362 | 0.738 |  | 0.671 | 0.747 | 0.592 | 0.652 | 0.697 | 0.344 | 0.731 |
| **T-CTD** | 0.662 | 0.719 | 0.606 | 0.645 | 0.680 | 0.327 | 0.721 |  | 0.657 | 0.696 | 0.618 | 0.651 | 0.673 | 0.314 | 0.723 |
| **D-CTD** | 0.713 | 0.828 | 0.597 | 0.672 | 0.742 | 0.438 | 0.780 |  | 0.713 | 0.818 | 0.606 | 0.680 | 0.742 | 0.434 | 0.784 |
| **AAindex** | 0.775 | 0.770 | 0.780 | 0.777 | 0.773 | 0.549 | 0.855 |  | 0.802 | 0.799 | 0.805 | 0.807 | 0.803 | 0.604 | 0.874 |
| **CTD** | 0.739 | 0.770 | 0.780 | 0.699 | 0.761 | 0.487 | 0.807 |  | 0.729 | 0.809 | 0.648 | 0.702 | 0.752 | 0.464 | 0.808 |
| **CTD+ESM** | 0.748 | 0.835 | 0.642 | 0.721 | 0.763 | 0.501 | 0.822 |  | 0.762 | 0.822 | 0.700 | 0.737 | 0.777 | 0.526 | 0.837 |
| **AAindex+ESM** | 0.777 | 0.810 | 0.687 | 0.772 | 0.778 | 0.553 | 0.857 |  | 0.790 | 0.781 | 0.800 | 0.800 | 0.790 | 0.581 | 0.867 |
| **AAindex+CTD** | 0.780 | 0.784 | 0.769 | 0.762 | 770 | 0.574 | 0.870 |  | 0.827 | 0.852 | 0.801 | 0.815 | 0.833 | 0.654 | 0.880 |
| **All features** | 0.788 | 0.832 | 0.743 | 0.764 | 0.797 | 0.578 | 0.869 |  | 0.807 | 0.839 | 0.774 | 0.792 | 0.815 | 0.615 | 0.886 |

| **Table S10.** Comparison of LightGBM classification performance using different feature selection methods on the 10-fold cross-validation data. | | | | | | | | |
| --- | --- | --- | --- | --- | --- | --- | --- | --- |
| **Dataset** | **Feature selection** | **ACC** | **SN** | **SP** | **PR** | **F1** | **MCC** | **AUC** |
| *H. sapiens* | Original features | 0.791 | 0.829 | 0.754 | 0.770 | 0.798 | 0.584 | 0.874 |
|  | ANOVA | 0.786 | 0.824 | 0.763 | 0.766 | 0.794 | 0.574 | 0.869 |
|  | RFE | 0.793 | 0.829 | 0.761 | 0.773 | 0.800 | 0.588 | 0.876 |
|  | LASSO | 0.782 | 0.812 | 0.766 | 0.765 | 0.788 | 0.565 | 0.867 |
|  | Elastic Net | 0.794 | 0.829 | 0.777 | 0.774 | 0.800 | 0.589 | 0.875 |
|  | MI | 0.791 | 0.831 | 0.748 | 0.770 | 0.799 | 0.522 | 0.876 |
| *T. gondii* | Original features | 0.766 | 0.784 | 0.748 | 0.758 | 0.771 | 0.533 | 0.852 |
|  | ANOVA | 0.754 | 0.777 | 0.732 | 0.743 | 0.760 | 0.508 | 0.847 |
|  | RFE | 0.773 | 0.793 | 0.753 | 0.763 | 0.778 | 0.546 | 0.858 |
|  | LASSO | 0.753 | 0.755 | 0.752 | 0.753 | 0.754 | 0.507 | 0.839 |
|  | Elastic Net | 0.768 | 0.787 | 0.750 | 0.760 | 0.773 | 0.537 | 0.852 |
|  | MI | 0.769 | 0.788 | 0.750 | 0.760 | 0.774 | 0.538 | 0.853 |
| *O. sativa* | Original features | 0.760 | 0.806 | 0.714 | 0.737 | 0.770 | 0.522 | 0.838 |
|  | ANOVA | 0.759 | 0.796 | 0.716 | 0.735 | 0.765 | 0.514 | 0.833 |
|  | RFE | 0.759 | 0.802 | 0.716 | 0.737 | 0.768 | 0.519 | 0.841 |
|  | LASSO | 0.754 | 0.791 | 0.718 | 0.736 | 0.762 | 0.510 | 0.833 |
|  | Elastic Net | 0.764 | 0.802 | 0.727 | 0.744 | 0.772 | 0.530 | 0.835 |
|  | MI | 0.761 | 0.803 | 0.719 | 0.739 | 0.770 | 0.524 | 0.841 |

| **Table S11.** Comparison of LightGBM classification performance using different feature selection methods on the test set. | | | | | | | | |
| --- | --- | --- | --- | --- | --- | --- | --- | --- |
| **Dataset** | **Feature selection method** | **ACC** | **SN** | **SP** | **PR** | **F1** | **MCC** | **AUC** |
| *H. sapiens* | Original features | 0.814 | 0.840 | 0.766 | 0.802 | 0.821 | 0.629 | 0.890 |
|  | ANOVA | 0.801 | 0.836 | 0.773 | 0.785 | 0.810 | 0.604 | **0.893** |
|  | RFE | 0.799 | 0.827 | 0.772 | 0.788 | 0.807 | 0.599 | 0.891 |
|  | LASSO | 0.811 | 0.831 | 0.789 | 0.802 | 0.816 | 0.621 | 0.889 |
|  | Elastic Net | 0.804 | 0.827 | 0.780 | 0.794 | 0.809 | 0.608 | 0.888 |
|  | MI | 0.816 | 0.836 | 0.787 | 0.793 | 0.813 | 0.610 | **0.893** |
| *T. gondii* | Original features | 0.781 | 0.799 | 0.763 | 0.764 | 0.781 | 0.562 | 0.867 |
|  | ANOVA | 0.774 | 0.789 | 0.763 | 0.761 | 0.773 | 0.548 | 0.865 |
|  | RFE | 0.779 | 0.797 | 0.761 | 0.762 | 0.779 | 0.559 | 0.874 |
|  | LASSO | 0.759 | 0.751 | 0.766 | 0.755 | 0.753 | 0.517 | 0.851 |
|  | Elastic Net | 0.782 | 0.786 | 0.777 | 0.773 | 0.779 | 0.563 | 0.866 |
|  | MI | 0.782 | 0.803 | 0.748 | 0.754 | 0.778 | 0.551 | **0.876** |
| *O. sativa* | Original features | 0.764 | 0.788 | 0.722 | 0.764 | 0.776 | 0.526 | 0.835 |
|  | ANOVA | 0.753 | 0.788 | 0.716 | 0.749 | 0.768 | 0.505 | 0.824 |
|  | RFE | 0.751 | 0.780 | 0.719 | 0.749 | 0.765 | 0.500 | 0.832 |
|  | LASSO | 0.757 | 0.780 | 0.731 | 0.759 | 0.769 | 0.513 | 0.829 |
|  | Elastic Net | 0.750 | 0.775 | 0.741 | 0.763 | 0.769 | 0.505 | 0.833 |
|  | MI | 0.765 | 0.782 | 0.737 | 0.752 | 0.767 | 0.517 | **0.847** |

| **Table S12.** Performance comparison of various machine learning algorithms on the validation data. | | | | | | | | |
| --- | --- | --- | --- | --- | --- | --- | --- | --- |
| **Dataset** | **Feature selection** | **ACC** | **SN** | **SP** | **PR** | **F1** | **MCC** | **AUC** |
| *H. sapiens* | KNN | 0.691 | 0.719 | 0.662 | 0.679 | 0.699 | 0.382 | 0.757 |
|  | AdaBoost | 0.750 | 0.773 | 0.727 | 0.739 | 0.755 | 0.501 | 0.827 |
|  | RF | 0.751 | 0.808 | 0.693 | 0.724 | 0.764 | 0.505 | 0.828 |
|  | SVM | 0.756 | 0.777 | 0.736 | 0.746 | 0.761 | 0.513 | 0.838 |
|  | XGBoost | 0.780 | 0.832 | 0.728 | 0.753 | 0.791 | 0.563 | 0.862 |
|  | CatBoost | 0.788 | 0.832 | 0.743 | 0.764 | 0.797 | 0.578 | 0.869 |
|  | LightGBM | **0.791** | **0.831** | **0.752** | **0.770** | **0.799** | **0.585** | **0.876** |
| *T. gondii* | KNN | 0.673 | 0.745 | 0.600 | 0.652 | 0.695 | 0.349 | 0.742 |
|  | AdaBoost | 0.742 | 0.754 | 0.730 | 0.737 | 0.746 | 0.484 | 0.820 |
|  | RF | 0.729 | 0.765 | 0.692 | 0.714 | 0.738 | 0.458 | 0.811 |
|  | SVM | 0.746 | 0.753 | 0.738 | 0.743 | 0.748 | 0.491 | 0.827 |
|  | XGBoost | 0.758 | 0.787 | 0.728 | 0.744 | 0.765 | 0.516 | 0.844 |
|  | CatBoost | 0.769 | 0.802 | 0.735 | 0.753 | 0.777 | 0.539 | 0.855 |
|  | LightGBM | **0.769** | **0.788** | **0.750** | **0.760** | **0.774** | **0.538** | **0.853** |
| *O. sativa* | KNN | 0.647 | 0.692 | 0.601 | 0.633 | 0.661 | 0.295 | 0.705 |
|  | AdaBoost | 0.720 | 0.737 | 0.704 | 0.711 | 0.724 | 0.441 | 0.791 |
|  | RF | 0.725 | 0.764 | 0.687 | 0.708 | 0.735 | 0.452 | 0.799 |
|  | SVM | 0.727 | 0.743 | 0.711 | 0.719 | 0.731 | 0.455 | 0.799 |
|  | XGBoost | 0.749 | 0.803 | 0.697 | 0.724 | 0.762 | 0.503 | 0.824 |
|  | CatBoost | 0.757 | 0.814 | 0.701 | 0.730 | 0.769 | 0.518 | 0.837 |
|  | LightGBM | **0.761** | **0.803** | **0.719** | **0.739** | **0.770** | **0.524** | **0.835** |

| **Table S13.** Performance comparison of various machine learning algorithms on the test set. | | | | | | | | |
| --- | --- | --- | --- | --- | --- | --- | --- | --- |
| **Dataset** | **Classifier** | **ACC** | **SN** | **SP** | **PR** | **F1** | **MCC** | **AUC** |
| *H. sapiens* | KNN | 0.704 | 0.733 | 0.677 | 0.697 | 0.714 | 0.407 | 0.776 |
|  | AdaBoost | 0.769 | 0.795 | 0.742 | 0.759 | 0.777 | 0.538 | 0.840 |
|  | RF | 0.762 | 0.813 | 0.709 | 0.741 | 0.776 | 0.525 | 0.843 |
|  | SVM | 0.762 | 0.770 | 0.753 | 0.762 | 0.766 | 0.523 | 0.848 |
|  | XGBoost | 0.793 | 0.837 | 0.748 | 0.773 | 0.804 | 0.588 | 0.878 |
|  | CatBoost | 0.807 | 0.839 | 0.774 | 0.792 | 0.815 | 0.615 | 0.886 |
|  | LightGBM | **0.816** | 0.836 | 0.766 | 0.793 | 0.813 | 0.610 | **0.893** |
| *T. gondii* | KNN | 0.680 | 0.755 | 0.608 | 0.649 | 0.698 | 0.366 | 0.751 |
|  | AdaBoost | 0.752 | 0.762 | 0.743 | 0.739 | 0.751 | 0.504 | 0.836 |
|  | RF | 0.754 | 0.784 | 0.724 | 0.732 | 0.757 | 0.509 | 0.831 |
|  | SVM | 0.766 | 0.777 | 0.756 | 0.754 | 0.765 | 0.533 | 0.841 |
|  | XGBoost | 0.776 | 0.803 | 0.749 | 0.755 | 0.778 | 0.553 | 0.864 |
|  | CatBoost | 0.774 | 0.815 | 0.734 | 0.747 | 0.779 | 0.551 | 0.868 |
|  | LightGBM | **0.782** | 0.803 | 0.748 | 0.754 | 0.778 | 0.551 | **0.876** |
| *O. sativa* | KNN | 0.638 | 0.662 | 0.613 | 0.648 | 0.655 | 0.275 | 0.693 |
|  | AdaBoost | 0.721 | 0.741 | 0.700 | 0.726 | 0.734 | 0.440 | 0.784 |
|  | RF | 0.726 | 0.747 | 0.704 | 0.731 | 0.739 | 0.452 | 0.787 |
|  | SVM | 0.727 | 0.730 | 0.724 | 0.741 | 0.735 | 0.454 | 0.804 |
|  | XGBoost | 0.747 | 0.782 | 0.709 | 0.743 | 0.762 | 0.492 | 0.821 |
|  | CatBoost | 0.759 | 0.789 | 0.727 | 0.758 | 0.773 | 0.518 | 0.833 |
|  | LightGBM | **0.765** | 0.782 | 0.722 | 0.752 | 0.767 | 0.517 | **0.847** |

| **Table S14.** Comparative performance evaluation of the proposed method against existing prediction models. | | | | | | | | |
| --- | --- | --- | --- | --- | --- | --- | --- | --- |
| **Data** | **Method** | **ACC** | **SN** | **SP** | **PR** | **F1** | **MCC** | **AUC** |
| *H.sapiens* | iLys-Khib | 0.703 | 0.739 | 0.665 | 0.697 | 0.717 | 0.405 | 0.774 |
|  | Khibpred | 0.709 | 0.699 | 0.720 | 0.722 | 0.710 | 0.419 | 0.781 |
|  | DeepKhib | 0.769 | 0.888 | 0.680 | 0.720 | 0.795 | 0.552 | 0.795 |
|  | ResNetKhib | 0.793 | 0.807 | 0.769 | 0.788 | 0.798 | 0.585 | 0.875 |
|  | HyLightKhib | **0.816** | **0.836** | **0.766** | **0.793** | **0.818** | **0.610** | **0.893** |
| *T-gondii* | iLys-Khib | 0.714 | 0.772 | 0.657 | 0.693 | 0.730 | 0.432 | 0.789 |
|  | Khibpred | 0.718 | 0.763 | 0.673 | 0.701 | 0.731 | 0.438 | 0.780 |
|  | DeepKhib | 0.772 | 0.812 | 0.736 | 0.746 | 0.777 | 0.547 | 0.837 |
|  | ResNetKhib | 0.777 | 0.786 | 0.771 | 0.765 | 0.775 | 0.553 | 0.852 |
|  | HyLightKhib | **0.782** | **0.803** | **0.748** | **0.754** | **0.778** | **0.551** | **0.876** |
| *O.sativa* | iLys-Khib | 0.672 | 0.734 | 0.606 | 0.660 | 0.695 | 0.344 | 0.729 |
|  | Khibpred | 0.687 | 0.677 | 0.696 | 0.670 | 0.688 | 0.373 | 0.751 |
|  | DeepKhib | 0.743 | 0.766 | 0.687 | 0.746 | 0.758 | 0.486 | 0.819 |
|  | ResNetKhib | 0.730 | 0.764 | 0.761 | 0.728 | 0.746 | 0.458 | 0.817 |
|  | HyLightKhib | **0.765** | **0.782** | **0.722** | **0.752** | **0.767** | **0.517** | **0.847** |
